# Multi-Omic Analysis Reveals Genetic Determinants and Therapeutic Targets of Chronic Kidney Disease and Kidney Function

**DOI:** 10.3390/ijms25116033

**Published:** 2024-05-30

**Authors:** Yao-Qi Lu, Yirong Wang

**Affiliations:** School of Biology & Basic Medical Sciences, Suzhou Medical College of Soochow University, Suzhou 215123, China; yaoqilu16@163.com

**Keywords:** chronic kidney disease, kidney function, multi-omic analysis, genomics, transcriptomics, proteomics, drug target, Mendelian randomization, inflammatory proteins, Golgi apparatus-related genes

## Abstract

Chronic kidney disease (CKD) presents a significant global health challenge, characterized by complex pathophysiology. This study utilized a multi-omic approach, integrating genomic data from the CKDGen consortium alongside transcriptomic, metabolomic, and proteomic data to elucidate the genetic underpinnings and identify therapeutic targets for CKD and kidney function. We employed a range of analytical methods including cross-tissue transcriptome-wide association studies (TWASs), Mendelian randomization (MR), summary-based MR (SMR), and molecular docking. These analyses collectively identified 146 cross-tissue genetic associations with CKD and kidney function. Key Golgi apparatus-related genes (GARGs) and 41 potential drug targets were highlighted, with MAP3K11 emerging as a significant gene from the TWAS and MR data, underscoring its potential as a therapeutic target. Capsaicin displayed promising drug–target interactions in molecular docking analyses. Additionally, metabolome- and proteome-wide MR (PWMR) analyses revealed 33 unique metabolites and critical inflammatory proteins such as FGF5 that are significantly linked to and colocalized with CKD and kidney function. These insights deepen our understanding of CKD pathogenesis and highlight novel targets for treatment and prevention.

## 1. Introduction

CKD has emerged as a leading global cause of death, with one of the most significant increases in mortality rates among all diseases over the past decade [1,2]. The complex etiology of CKD, driven by various genetic and environmental factors, complicates understanding its pathophysiology [3]. Therefore, identifying the genetic determinants of CKD and kidney function is crucial for developing prevention strategies, discovering novel therapeutic targets, and implementing precision medicine strategies to improve patient outcomes.

Recent advances in genome-wide association studies (GWASs) from the published projects of the CKDGen Consortium have unveiled numerous genetic variants associated with CKD and kidney function-related traits, including biomarkers used to quantify kidney function, such as glomerular filtration rate estimated from creatinine levels (eGFRcrea), glomerular filtration rate estimated from serum cystatin C levels (eGFRcys), and blood urea nitrogen (BUN), as well as the urine albumin-to-creatinine ratio (UACR), which serves as a measure of kidney damage [4,5,6]. However, the mechanisms by which these loci influence CKD and kidney function progression remain largely unelucidated, primarily because most GWAS findings reside in non-coding regions of the genome, making them difficult to interpret. TWAS offers an effective solution to overcome this limitation by focusing on genes that are more interpretable and functional units than variants [7,8,9]. Nonetheless, the effectiveness of TWAS may be diminished by small sample sizes in expression quantitative trait locus (eQTL) data or the absence of data from causally relevant tissues [10]. Recent research suggests that gene regulation is often conserved across tissues [11,12,13]. Therefore, integrating eQTL data from multiple tissues can significantly enhance the power of TWAS in complex traits, such as CKD [14]. Additionally, MR and SMR have emerged as powerful approaches to establish risk factors and identify drug targets for diseases. Combining MR and SMR with multi-omic data offers valuable insights into disease etiology and potential therapeutic targets, enhancing our understanding of the genetic underpinnings of diseases [15,16,17].

**Figure 1:**
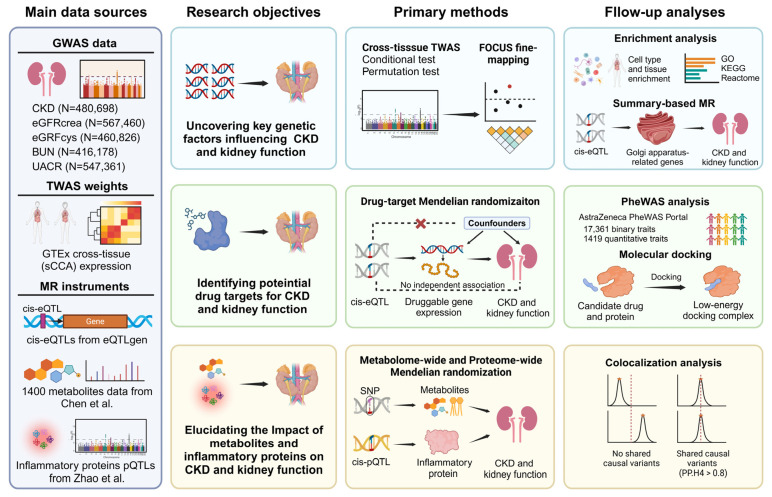
Study overview. An overview of this study’s data sources, analytical flow, and methodology. Created with BioRender.com. GWAS: genome-wide association study, TWAS: transcriptome-wide association study, FOCUS: fine-mapping of causal gene sets, eQTL: expression quantitative trait locus, pQTL: protein quantitative trait locus, PheWAS: phenome-wide association study, MR: Mendelian randomization, SMR: summary-based MR, PWMR: proteome-wide MR, sCCA: sparse canonical correlation analysis, PP.H4: posterior probability of hypothesis 4.

Our study employed a comprehensive multi-omic approach to uncover the genetic architecture of CKD and kidney function and identify potential therapeutic targets (Figure 1 displays a study overview). Using TWAS, followed by fine-mapping and conditional analyses, we identified high-confidence genes crucial to CKD and kidney function. Further enrichment and SMR analyses shed light on the significant role of the Golgi apparatus in CKD and kidney function, unveiling a potential therapeutic pathway. Additionally, drug–target MR analysis identified promising therapeutic targets, with subsequent drug prediction and molecular docking studies indicating effective drug–target interactions. Metabolome-wide and proteome-wide MR analyses revealed significant associations among CKD, kidney function, and specific metabolites and inflammatory proteins, suggesting potential therapeutic pathways. In summary, our research facilitates the understanding of the genetic underpinnings of CKD and kidney function, identifying promising therapeutic targets and offering novel insights for CKD management and treatment.

## 2. Results

### 2.1. TWASs Identify Key Genetic Determinants of CKD and Kidney Function

**Figure 2:**
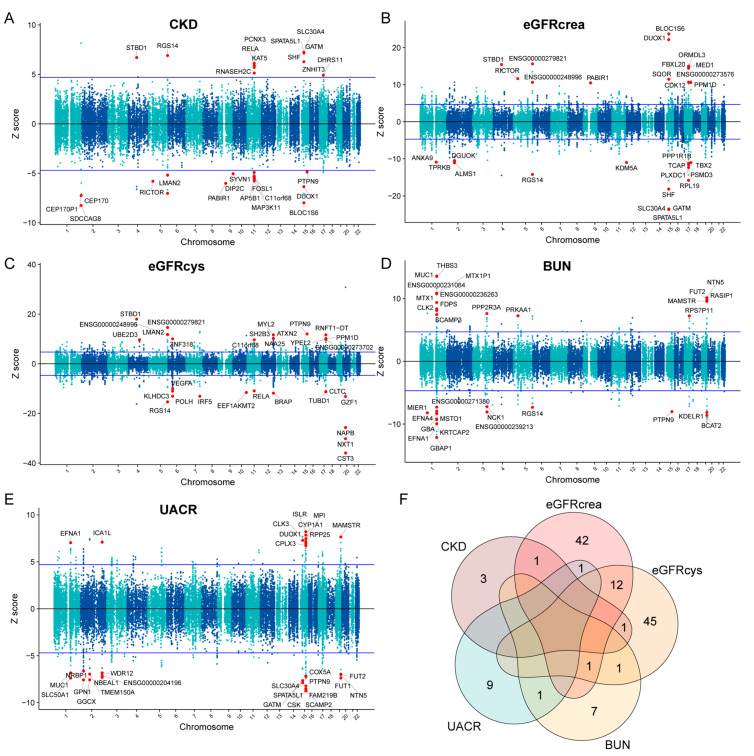
TWAS results for CKD and kidney function. (**A**–**E**). Manhattan plots of gene–trait associations for CKD, eGFRcrea, eGFRcys, BUN, and UACR. The *X*-axis represents genomic positions, and the *Y*−axis represents the Z-scores of the gene–trait associations. The blue lines indicate the Bonferroni-corrected significance threshold (Z = 4.837, corresponding to *p* = 1.32 × 10^−6^). The top 30 statistically significant gene-trait associations are highlighted with red circles. (**F**). Venn diagram illustrating the overlap of the 146 significant high-confidence TWAS features identified through the integration of TWAS, colocalization, permutation analysis, and FOCUS fine-mapping. The numbers within the diagram represent the count of significant features specific to each trait or shared among multiple traits.

We used FUSION [18], cross-tissue eQTL weights [14], and GWAS summary statistics to impute gene expression signatures associated with CKD and kidney function-related traits, including eGFRcrea, eGFRcys, BUN, and UACR. Our extensive TWAS results, documented in Supplementary Tables S1–S8, incorporate results of colocalization analyses and permutation tests (Supplementary Tables S1–S5), conditional analyses (Supplementary Table S6), and fine-mapping of causal gene sets (FOCUS) analysis [19] (Supplementary Table S7). Our analysis delineated 28 cross-tissue features significantly associated with CKD, 565 with eGFRcrea, 488 with eGFRcys, 100 with BUN, and 142 with UACR, all surpassing the Bonferroni threshold of *p* < 1.32 × 10^−6^ (Figure 2). To address potential bias from linkage disequilibrium (LD) among variants affecting gene expression and phenotypes independently, we conducted colocalization analyses [20]. This analysis affirmed that nearly half of the associations were underpinned by shared pleiotropic single-nucleotide polymorphisms (SNPs), impacting both gene expression and respective phenotypes (10/28 for CKD, 203/565 for eGFRcrea, 189/488 for eGFRcys, 40/100 for BUN, and 61/142 for UACR). Furthermore, many of these significant findings were confirmed using strict permutation testing, indicating that they are likely true effects rather than spurious findings linked to strong GWAS signals. Additionally, 14 CKD, 220 eGFRcrea, 164 eGFRcys, 28 BUN, and 41 UACR unique features passed conditional analyses, suggesting genes that directly influence kidney function-related traits rather than those co-expressed due to shared genetic factors in the same region. FOCUS fine-mapping subsequently pinpointed potential causal genes with high confidence. Ultimately, we identified five high-confidence features for CKD, 60 for eGFRcrea, 60 for eGFRcys, 10 for BUN, and 11 for UACR (Table 1 and Supplementary Table S8). Notably, 12 features shared between eGFRcrea and eGFRcys likely reflect general kidney function attributes rather than specific to creatinine or cystatin C metabolism (Figure 2). Among the five high-confidence features for CKD, DIP2C, and RICTOR were each observed in eGFRcrea and eGFRcys, underscoring their critical role in kidney health.

### 2.2. Cell-Type and Tissue Enrichment Analysis for High-Confidence TWAS Genes

Using the CellMarker database through Enrichr, we analyzed the cell and tissue expression specificity of the high-confidence genes identified from our TWAS analysis. The results indicate significant enrichment of these genes in several cell and tissue types (Supplementary Figure S1). Notably, we observed significant enrichment in natural killer T (NKT) cells from fetal kidney based on 45 high-confidence genes that are highly expressed in this cell type. The significant enrichment of these genes in NKT cells from fetal kidney suggests that they may play an essential role in the early development and immune modulation of the kidney. Additionally, the enrichment of significant genes in other cell and tissue types indicates the functional diversity of these genes and the complex genetic underpinnings of CKD and kidney function.

### 2.3. High-Confidence TWAS Genes Are Associated with Diverse Biological Functions

**Figure 3:**
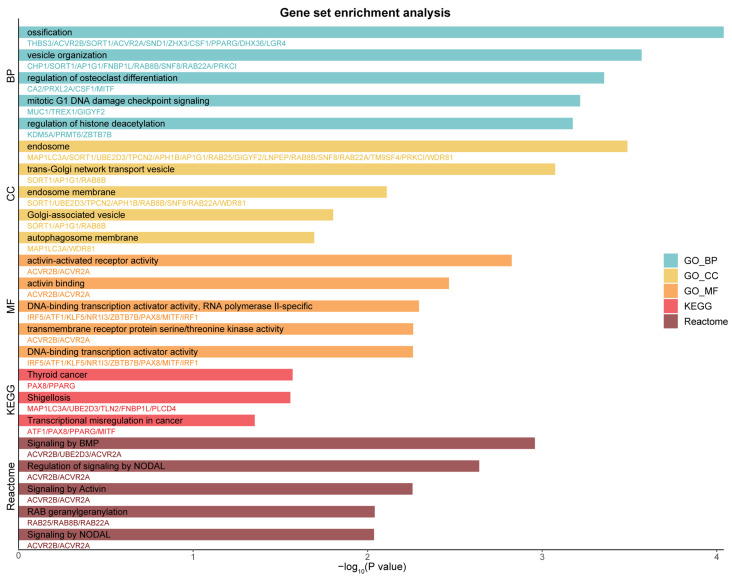
Gene set enrichment analysis for high-confidence TWAS genes. The bar plot displays the top 5 enriched gene sets in the study, categorized by biological process (BP), cellular component (CC), molecular function (MF), KEGG pathways, and Reactome pathways. Alongside each bar, the associated genes for the corresponding terms are specified.

To investigate the function of the high-confidence genes identified through the TWAS, we conducted a comprehensive gene set enrichment analysis. The analysis revealed that our high-confidence genes are associated with a broad range of biological processes, cellular components, and molecular functions (Figure 3). Specifically, biological processes, including ossification and vesicle organization, were found to be significantly enriched, suggesting that these genes play significant roles in skeletal health and intracellular transport. In terms of cellular components, notable enrichment in components such as the endosome and Golgi apparatus underscores these genes’ vital roles in cellular trafficking and processing. Moreover, molecular functions like activin-activated receptor activity were prominently enriched, reflecting their potential involvement in signaling pathways. Kyoto Encyclopedia of Genes and Genomes (KEGG) pathway analysis revealed the significant engagement of these genes in pathways related to thyroid cancer, shigellosis, and transcriptional misregulation in cancer. Additionally, Reactome pathway analysis identified significant associations in pathways like signaling by bone morphogenetic protein (BMP), RAB geranylgeranylation, and signaling by activin, which are closely associated with the TGF-β signaling pathway [21,22,23]. The TGF-β signaling pathway is intimately linked to kidney fibrosis and plays a critical role in the progression of CKD. These results underscore the complex genetic underpinnings of these genes.

The diverse functions of the identified genes may offer novel insights into renal pathophysiology and potential therapeutic targets. Notably, the enrichment analysis results included multiple gene sets associated with the Golgi apparatus (Figure 3), possibly indicating a close relationship between Golgi apparatus function and CKD as well as kidney function. Further investigation into this connection may reveal new genetic mechanisms and therapeutic targets.

### 2.4. SMR Identifies the Association of GARGs with CKD and Kidney Function

To further investigate the association of GARGs with CKD and kidney function, we conducted SMR analyses of GARGs on CKD and kidney-related traits. The results showing strong evidence of an association (false discovery rate (FDR)-adjusted *P*_SMR_ < 0.1) were assessed using the heterogeneity independent instruments (HEIDI) test (*P*_HEIDI_ > 0.01), which was implemented with SMR software (version 1.3.1). The HEIDI test was used to investigate whether the association was due to a shared causal variant or pleiotropy. We thus identified two unique genetic associations for CKD, 62 for eGFRcrea, 74 for eGFRcys, 22 for BUN, and 32 for UACR. Sensitivity analysis using additional MR methods relying on similar assumptions was conducted and shown to support our findings (Supplementary Tables S9–S13). We further performed colocalization analysis to detect shared causal variants between the target trait and GARG eQTLs. Results indicated that 16 genes exhibited strong evidence of colocalization with the CKD and kidney-related traits (posterior probability of hypothesis 4 (PP.H4) > 0.80) (Table 2). Higher ATF6B expression levels were associated with an increased risk of CKD. Higher NFE2L2 expression was significantly linked to higher eGFRcrea. Conversely, higher STK24 and PRKCE expression levels were associated with lower eGFRcrea, with PRKCE additionally associated with lower eGFRcys, suggesting a potentially detrimental impact on kidney function. Moreover, higher RASIP1 expression was negatively associated with BUN levels and positively associated with UACR, illustrating its complex role in kidney health. These results highlight the significant impact of Golgi apparatus-related genes on CKD and kidney function.

### 2.5. MR Identifies Potential Drug Targets for CKD and Kidney Function

To identify additional potential therapeutic targets for CKD and kidney function, our study embarked on a drug–target MR analysis, in parallel with our TWAS. Leveraging eQTLs within 100 kb of each druggable gene as instrumental variables, we assessed their impact on CKD and related kidney function traits [24]. Our primary MR analysis identified six unique genetic targets for CKD, 58 for eGFRcrea, 61 for eGFRcys, 23 for BUN, and 31 for UACR, following Bonferroni correction (Supplementary Table S14).

However, sensitivity tests revealed that AKR1A1, DSTYK, GSR, MAPK3, PDIA3, and SLC22A5 demonstrated inconsistent effect directions across different MR methods (Supplementary Table S14), while BLK, PTGFR, NRG1, C12orf39, SLC22A5, and LTBP4 did not pass the horizontal pleiotropy assessment (*p* < 0.05, Supplementary Table S15). Subsequent colocalization analyses aimed to ascertain whether SNPs linked to both the target trait and eQTL share causal variants. Results indicated 34 genes exhibited substantial colocalization with the target traits (PP.H4 > 0.80), positioning them as candidate therapeutic targets (1/6 for CKD, 16/55 for eGFRcrea, 14/57 for eGFRcys, 6/22 for BUN, and 4/28 for UACR) (Table 3 and Supplementary Table S17). Notably, genes such as MAP3K11, ACVR2A, ITIH4, KLHL24, LNPEP, and THBS3 were confirmed as high-confidence genes from our TWAS, highlighting their potential critical roles in CKD and kidney function. In particular, MR results suggest that decreased MAP3K11 expression correlates with a lower CKD risk. Furthermore, CASP9 was significantly associated with eGFRcrea, eGFRcys, and BUN, which is consistent with previous reports of its role in renal pathologies [25].

### 2.6. Phenome-Wide Association Study (PheWAS) Analysis for MR-Identified Druggable Genes

To assess the implications of pharmacological intervention on genes identified using MR analysis, we conducted a PheWAS utilizing the AstraZeneca PheWAS Portal database [26]. Our findings, detailed in Supplementary Table S18, indicate that the majority of the druggable genes identified did not exhibit significant associations with non-renal traits. This lack of association suggests minimal off-target effects and horizontal pleiotropy, thus reinforcing the specificity and potential safety of these potential drug targets. Notably, eight genes were linked to 14 distinct phenotypes, with IMPDH2 and ITIH4 being specifically related to inflammatory traits, while LAMC1 and NEU1 were correlated with cardiometabolic traits. Given the intricate interplay among kidney disease, inflammation, and cardiometabolic processes, future research endeavors should prioritize investigating how the multifaceted roles of these genes may influence kidney disease therapeutics.

### 2.7. Candidate Drug Prediction

**Figure 4:**
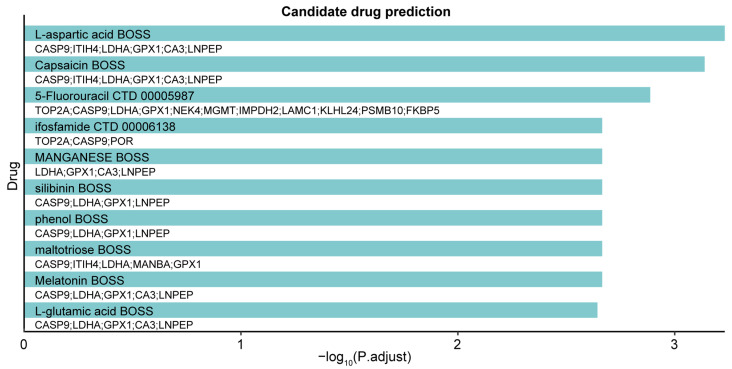
Candidate drug predicted for MR-identified druggable genes. This bar plot showcases the top 10 candidate drugs as determined using enrichment analysis with the DSigDB database. The target genes associated with each candidate drug are listed alongside their respective bars.

We utilized the DSigDB database to identify potentially effective intervention drugs for the druggable genes identified in the MR study. Based on the adjusted *p*-values, the top 10 potential chemical compounds were shown (Figure 4). The result showed that L-aspartic acid and capsaicin were the most significant drugs, linked to six druggable genes. Furthermore, 5-fluorouracil, a therapeutic agent for various cancers, showed the most extensive gene interactions.

### 2.8. Molecular Docking Analysis Unraveling Drug–Target Affinity and Evaluating Druggability

**Figure 5:**
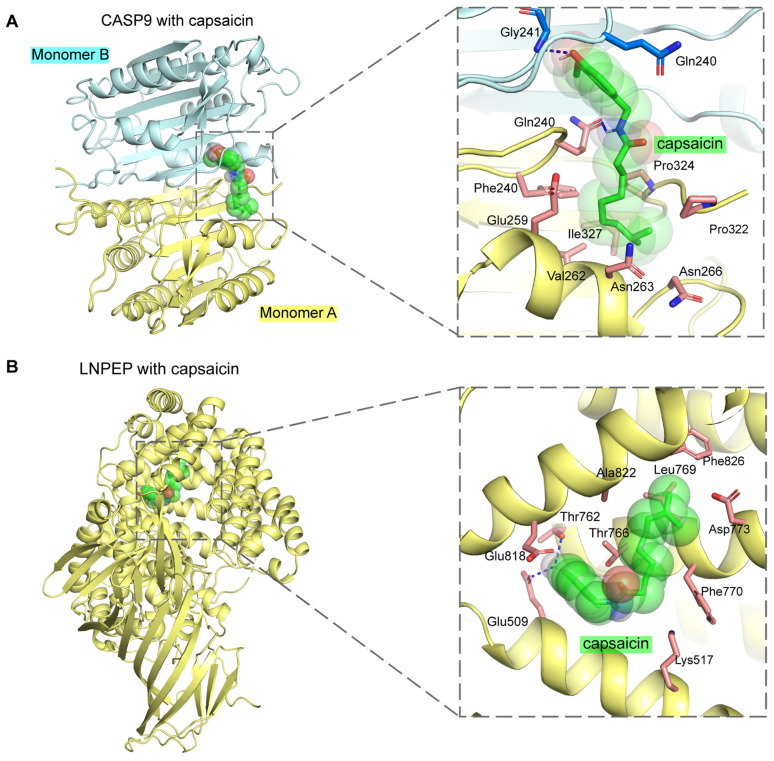
Molecular docking analysis of capsaicin with CASP9 and LNPEP. (**A**). (**Left**) The overall structure of the CASP9 dimer complexed with capsaicin, with monomer A depicted in yellow and monomer B in cyan. (**Right**) A close-up view of the binding pocket, with capsaicin highlighted in green, the amino acid residues involved in binding shown as pale red sticks, and polar interactions indicated by blue dashed lines. (**B**). (**Left**) The overall structure of LNPEP in complex with capsaicin. (**Right**) A close-up view of the binding pocket, with the color scheme and symbols corresponding to those in panel A.

To assess the potential druggability of targets by assessing the affinity of drug candidates, we performed molecular docking to explore the interactions between the top three drug candidates and the proteins encoded by the corresponding genes. The AutoDock Vina was utilized to conduct the molecular docking studies, and a total of 23 docking results were obtained, 17 of which had binding energies below −5.0 kcal/mol (Table 4, Figure 5 and Supplementary Figures S2–S16). Binding energy is a measure of the strength of the interaction between a drug and its target, with lower values indicating more stable binding. The interaction between capsaicin and CASP9, characterized by the lowest binding energy of −7.483 kcal/mol, signifies highly stable binding, highlighting its potential as a drug candidate. Specifically, capsaicin is situated at the dimer interface of CASP9, where it forms hydrogen bonds with Gly241 (Monomer B) and Gln240 (Monomer A), alongside hydrophobic interactions with several other residues, leading to a stable binding conformation (Figure 5A). Similarly, capsaicin forms both hydrophobic and hydrophilic interactions at the catalytic region and D4 domain interface of LNPEP, indicating a potential regulatory effect on LNPEP activity (Figure 5B). For all drug–target interactions with binding energies below −5.0 kcal/mol, the formation of hydrogen bonds and the achievement of stable conformations within the protein targets were consistently observed, highlighting the potential of these drug candidates (Supplementary Figures S2–S16).

### 2.9. PWMR Identifies Links between Inflammatory Proteins and Kidney Disease

Given the significant link between inflammation and CKD, as highlighted by our PheWAS findings, we performed a PWMR analysis of 91 circulating inflammatory proteins on CKD and kidney-related traits to further investigate their roles in CKD and kidney function. The primary MR analysis revealed significant associations with two for eGFRcys, two for BUN, and three for UACR after Bonferroni correction (Supplementary Tables S19–S23). However, sensitivity analyses indicated that SULT1A1 showed an inconsistent direction of effect across three methods (Supplementary Table S20). In addition, horizontal pleiotropy was not detected using MR-Egger intercept tests (*p* > 0.05), supporting the robustness of our results. The MR analysis highlighted the renoprotective potential of FGF5, with higher plasma FGF5 levels associated with a lower risk of CKD (OR = 0.908), higher eGFRcrea (β = 0.005) and eGFRcys (β = 0.004), and lower BUN levels (β = −0.007) (Table 5). Further colocalization analysis revealed that five of the 13 proteins exhibited strong evidence of colocalization with their respective target traits (PP.H4 > 0.8), especially FGF5, which demonstrated significant colocalization with CKD, eGFRcrea, eGFRcys, and BUN, reinforcing its potential as a therapeutic target for CKD.

### 2.10. Metabolome-Wide MR Reveals Key Metabolites Associated with CKD and Kidney Function

**Figure 6:**
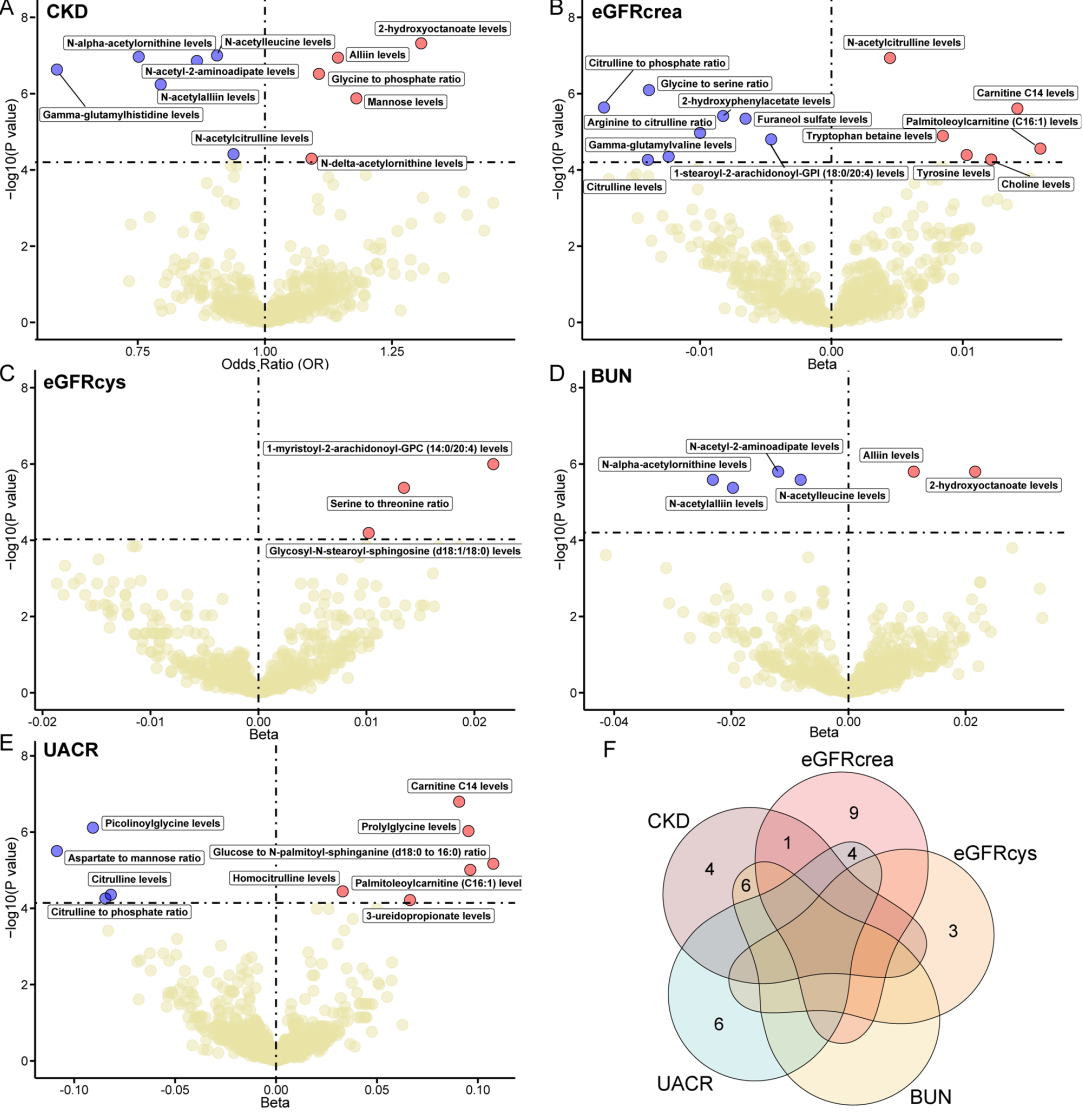
Results of metabolome-wide MR analysis on CKD and kidney function. (**A**–**E**). The volcano plots illustrate the association of metabolites with CKD (**A**), eGFRcrea (**B**), eGFRcys (**C**), BUN (**D**), and UACR (**E**). The effect size is plotted on the *X*-axis with odds ratio (OR) for CKD and beta coefficients for other kidney function traits. The *Y*-axis represents the −log_10_(*p*-value), quantifying the statistical significance of the metabolite associations. The dotted line corresponds to the Bonferroni-adjusted significance threshold, with metabolites above this line considered significant. Red points denote metabolites with a positive effect, blue points represent those with a negative effect, and yellow points signify non-significant associations. (**F**). Venn diagram illustrating the overlap of the 33 significant metabolites identified across the different kidney-related traits in the metabolome-wide MR. The numbers within the diagram represent the count of significant metabolites specific to each trait or shared among multiple traits.

To investigate the impact of circulating metabolites on CKD and kidney function, we performed a metabolome-wide MR analysis of 1091 blood metabolites and 309 metabolite ratios on CKD and kidney-related traits. The main analysis identified 34 metabolites or metabolite ratios with significant effects on CKD, 21 on eGFRcrea, three on eGFRcys, six on BUN, and 12 on UACR, after Bonferroni correction (Supplementary Table S24). No horizontal pleiotropy was detected using MR-Egger intercept tests (*p* > 0.05), affirming the robustness of our findings (Supplementary Table S25). Moreover, we eliminated genetic variants associated with multiple metabolites and unidentified metabolites. Ultimately, we identified a total of 33 metabolites that significantly impact CKD and kidney function-related phenotypes (Figure 6). Notably, 2-hydroxyoctanoate (OR = 1.30) and Gamma-glutamyl histidine (OR = 0.59) were the most positively and negatively correlated with CKD, respectively. Palmitoleoylcarnitine (C16:1) showed the highest positive association with eGFRcrea (β = 0.016), while the citrulline to phosphate ratio was most negatively correlated (β = −0.017). For eGFRcys, the metabolite 1-myristoyl-2-arachidonoyl-GPC (14:0/20:4) had the strongest positive correlation (β = 0.022). For BUN, 2-hydroxyoctanoate also showed a strong positive correlation (β = 0.022), whereas N-alpha-acetylornithine had a significant negative association (β = −0.023). Finally, for UACR, the glucose to N-palmitoyl-sphinganine (d18:0 to 16:0) ratio showed the highest positive correlation (β = 0.108), and the aspartate to mannose ratio emerged as the metabolite with the strongest negative correlation (β = −0.109). Additionally, several metabolites were identified as being significantly associated with multiple traits (Figure 6F). For example, 2-hydroxyoctanoate was linked closely with both CKD and BUN, while N-acetylcitrulline was significantly associated with CKD and eGFRcrea. Notably, higher levels of N-acetylalliin are associated with a lower risk of CKD and decreased BUN levels, highlighting its potential protective role in kidney health. These insights highlight the critical role of circulating metabolites in kidney health, emphasizing their importance in CKD research and the need to explore their mechanisms further.

## 3. Discussion

Our study represents a significant advancement in understanding the complex genetic underlying of CKD and kidney function by leveraging large genomic data sources and a multi-omic approach. To enhance the study’s reliability and comprehensiveness, we conducted comprehensive analyses across CKD, various kidney function markers (eGFRcrea, eGFRcys, and BUN), and an indicator of kidney damage (UACR). Our TWAS identified 146 high-confidence cross-tissue features closely linked to CKD and kidney function, shedding light on the intricate genetic framework influencing the disease. Further gene enrichment analysis revealed a significant association of these genes with various processes, such as bone health and the Golgi apparatus, underscoring the complex genetic basis of kidney function. Building on this foundation, we performed SMR analyses and identified several GARGs significantly associated with CKD and kidney function, thereby highlighting the critical role of GARGs in kidney pathology. In parallel, our drug–target MR analysis identified 34 potential therapeutic targets, notably MAP3K11 and CASP9, tied to causal relationships with CKD and kidney function, suggesting their viability as treatment targets. Furthermore, our drug prediction and molecular docking analysis identified potential compounds, like L-aspartic acid and capsaicin, indicating new pathways for CKD treatment through these targets. These findings offer promising avenues for the development of targeted therapies for CKD. Further expanding our understanding, the PWMR analysis highlighted the significance of FGF5 in CKD and kidney function, suggesting it as a potential therapeutic target. The metabolome-wide MR analysis also identified key metabolites associated with CKD and kidney function, such as 2-hydroxyoctanoate and N-acetylalliin, illustrating the critical role of circulating metabolites in kidney health. Overall, our study illuminates the complex genetic, metabolic, and inflammatory underpinnings of CKD and kidney function, providing novel insights into its complex etiology and identifying promising therapeutic targets for future research and drug development.

Our TWAS identified many genes that have been previously reported to be closely associated with kidney function and disease, supporting the validity of our approach. For instance, the expression level RICTOR is significantly associated with a decreased risk of CKD and an increased level of eGFRcys, corroborating previous studies that highlighted its crucial role in protecting against kidney diseases, including acute kidney injury and renal inflammation [27,28]. IRF5 significantly influences the progression of polycystic kidney disease (PKD) by enhancing inflammatory cytokine production in resident macrophages, a key factor in accelerating cystogenesis [29]. L3MBTL2 plays a protective role in kidney injury, partly by inhibiting the DNA damage-p53-apoptosis pathway [30]. Specifically, carboxy-terminal frameshift variants in TREX1 mutations have been associated with autosomal dominant renal thrombotic microangiopathy (TMA) and CKD [31]. SH3YL1 protein plays a crucial role in mediating oxidative stress pathways leading to acute kidney injury and serves as a predictive biomarker for renal outcomes in type 2 diabetes patients [32]. KLF5 contributes to the preservation of podocyte function and prevention of apoptosis through the regulation of crucial pathways, which are pivotal in the context of kidney diseases [33].

Among the 60 unique high-confidence TWAS genes associated with eGFRcrea, 20 overlapped with loci previously reported in the GWAS study by Stanzick KJ et al., who identified 424 non-overlapping loci for eGFRcrea [5]. This overlap underscores the robustness of our findings and suggests that these genes play significant roles in kidney function, as evidenced by their identification using both GWAS and TWAS methodologies. The methodological differences between TWASs and GWASs contribute to the complementary nature of these findings. While GWASs identify loci associated with traits, TWASs link these loci to specific gene expressions, offering a more functional understanding of genetic associations and highlighting regulatory mechanisms.

The cell type and tissue enrichment analysis revealed significant enrichment of 45 high-confidence TWAS genes in natural killer T (NKT) cells from fetal kidney. NKT cells play crucial roles in kidney diseases, influencing inflammation, tissue repair, and fibrosis. The identified genes may modulate immune responses, contributing to renal pathophysiology. Previous research has shown that NKT cells in the kidney increase with age and can be further activated by synthetic ligands (alpha-galactosylceramide) or bacterial DNA (CpG-ODN), causing acute kidney injury (AKI). They also play significant roles in lupus nephritis through Th2 immune responses [34]. In summary, our findings of significant gene enrichment in NKT cells from fetal kidney add a new dimension to the existing understanding of NKT cells’ roles in kidney health and disease, potentially highlighting new pathways for therapeutic intervention.

Our gene set enrichment analysis identified three TGF-β family members—ACVR2A, ACVR2B, and UBE2D3—and three Rab family members—RAB25, RAB8B, and RAB22A—that are closely associated with the TGF-β signaling pathway [23]. Kidney fibrosis, a hallmark of CKD progression, is characterized by the excessive accumulation of extracellular matrix (ECM) components, leading to scarring and functional decline of the kidney [35]. The TGF-β signaling pathway plays a central role in this process by promoting the activation of fibroblasts and the production of ECM proteins. Specifically, TGF-β1 drives epithelial-mesenchymal transition (EMT) and ECM deposition in the kidney [36]. Accordingly, these six genes may play significant roles in kidney fibrosis, and further experimental investigation into their roles in the onset and progression of kidney disease is warranted.

Additionally, our gene set enrichment analysis identified the high-confidence TWAS genes significantly associated with vesicle organization, endosome, trans-Golgi network transport vesicle, and Golgi-associated vesicles. These processes are closely linked to the Golgi apparatus, suggesting a potentially significant relationship between the function of the Golgi apparatus and both CKD and kidney function. The Golgi apparatus is crucial for a variety of cellular homeostatic functions, including trafficking, sorting, and modification of proteins and lipids. Dysfunctions in Golgi-associated processes have been implicated in a wide range of diseases, including neurodegenerative disorders, cancer, infectious diseases, and cardiovascular conditions [37]. Prompted by these insights, we conducted SMR analysis to explore the role of GARGs in CKD and kidney function, identifying several GARGs, such as NFE2L2, STK24, and PRKCE, with significant associations with CKD and kidney function. Our study demonstrated that increased PRKCE expression levels correlate with lower eGFRcrea, while recent research suggests that inhibiting PKC-ε signaling could prevent hypoxia-induced acute kidney injury (AKI), indicating that suppressing PRKCE expression might offer renal protective effects [38]. Additionally, recent research has highlighted the role of the Pdcd10–STK24/25 complex in controlling kidney water reabsorption, underscoring the importance of STK24 in kidney function [39]. Notably, NRF2, a transcription factor encoded by NFE2L2, plays a critical role in regulating redox balance and antioxidant responses, offering protection against oxidative stress and inflammation in the kidney [40]. Several studies have demonstrated the protective roles of NRF2 activation in various kidney diseases, including acute kidney injury, diabetic nephropathy, and CKD, making it a promising therapeutic target for kidney diseases [41,42,43].

Our drug–target MR analysis underscored key genes that could serve as therapeutic targets for CKD and kidney function. MAP3K11, with a strong positive association with CKD supported by colocalization evidence, was also identified as a high-confidence gene using TWAS. Additionally, members of the MAP3K family, such as MAP3K5 (ASK1) and MAP3K14 (NIK), are known to be important in kidney disease [44], suggesting that modulating MAP3K11 activity could be a promising therapeutic strategy. The association between GPX1 and decreased eGFRcrea highlights its critical role in kidney disease progression. As a vital enzyme that neutralizes hydrogen peroxide, GPX1 is essential in protecting cells from oxidative damage [45]. Previous research suggests that GPX1 could act as both a biomarker and a therapeutic target for renal cell carcinoma (RCC) [46]. This insight positions GPX1 as a promising therapeutic target for CKD, underscoring the tight connection between oxidative balance and kidney health. NRG4 shows a strong association with preserved kidney function, alongside reduced mRNA expression in the adipose tissue of diabetic kidney disease (DKD) mice and a positive impact on glucose and lipid profiles [47]. This underscores its dual therapeutic potential in tackling both metabolic and renal diseases, emphasizing the intricate connection between kidney health and metabolic regulation. Our analysis also identified a significant association between CASP9 genetic variants and eGFR as well as BUN. Consistent with this, previous research by Doke et al. demonstrated that CASP9 plays a crucial role in CKD pathogenesis, implicating apoptosis, mitophagy, and inflammation in kidney damage [25], thus highlighting the potential of CASP9 inhibition in preserving kidney function and offering a new avenue for CKD treatment development. Furthermore, we identified several potential drug targets, such as L-aspartic acid, capsaicin, and 5-fluorouracil (Figure 4). Remarkably, capsaicin has shown therapeutic potential in managing kidney diseases attributed to its diverse biological actions [48]. Experimental evidence suggests that capsaicin may slow the progression of CKD by inhibiting key fibrotic signaling pathways, notably TGF-β1 and Smad2/3, and by activating the TRPV1 channel [49,50,51,52]. Moreover, molecular docking analysis demonstrated that the interaction between capsaicin and CASP9, with the lowest binding energy of −7.483 kcal/mol, enhances its potential as a drug target (Table 4 and Figure 5A). Based on our findings and corroborating evidence from previous studies, we propose that MAP3K11 and CASP9 be prioritized as key candidates for future experimental and clinical research.

Inflammation plays a critical role in the development and progression of CKD [53]. Our results reveal a correlation between higher FGF5 levels and a lower risk of CKD, improvements in eGFR, and decreased BUN levels, underscoring its potential for renal protection. Through our analysis, FGF5 has been identified as a key factor in managing CKD and enhancing kidney health. Previous studies have established a relationship between higher expression of FGF5 and increased eGFR [54,55]. Furthermore, recent research highlights the ability of FGF5 to mitigate inflammation. For instance, FGF5 demonstrates a protective effect against liver injury by diminishing liver inflammation [56]. In addition, overexpression of FGF5 mitigates inflammation, oxidative stress, and spinal cord injury through AMPK activation [57]. Given these findings, we hypothesize that FGF5 may confer protection to kidney by alleviating inflammation. Our PWMR analyses, identifying genetic loci and inflammatory proteins associated with CKD and kidney function, further underscore the crucial influence of inflammation on kidney health and the potential of targeted anti-inflammatory therapies in the management of CKD.

Our metabolome-wide MR analysis, leveraging the latest GWAS data on 1400 metabolites [58], identified 33 metabolic traits that significantly impact CKD and kidney function, highlighting the pivotal role of the plasma metabolome in kidney health. Notably, citrulline significantly correlates with eGFRcrea, and its derivative, N-acetylcitrulline, also strongly correlates with both eGFRcrea and CKD (Figure 6). These findings corroborate previous studies that identified citrulline supplementation as promoting an anti-inflammatory profile and nephron preservation, underscoring its vital roles in kidney health [59]. Furthermore, our study found that elevated levels of N-acetylalliin are associated with a lower risk of CKD and decreased BUN levels (Figure 6). These findings not only corroborate previous research that indicated a positive correlation between N-acetylalliin and increased eGFR levels but also expand upon our understanding of the crucial role of N-acetylalliin in kidney health [60]. Intriguingly, our results further reveal that alliin, a chemically similar compound, exerts opposite effects, with higher levels linked to an increased risk of CKD and elevated BUN levels. This observation underscores the notion that similar compounds can exhibit vastly divergent biological activities after undergoing distinct chemical modifications. The contrasting impacts of alliin and N-acetylalliin on kidney health emphasize the significance of chemical modifications in determining the biological activity of compounds, paving the way for future research into the potential therapeutic applications of chemically modified natural compounds for kidney disease and beyond.

Our research boasts several strengths, marking significant advancements in the study of CKD and kidney function. Key among these is the enrichment of large GWAS datasets with transcriptomic imputation, leveraging cross-tissue gene expression weights created using sparse canonical correlation analysis (sCCA) to enhance the statistical power of our TWAS. This approach not only facilitated the identification of key genetic factors related to CKD and kidney function but also unveiled the disease’s complex, cross-tissue characteristics. Employing the FUSION pipeline, we distinguished causal gene–trait associations from correlations that merely reflect large GWAS signals or LD, enhancing the precision of our results. Further refinement was achieved through FOCUS fine-mapping, which identified putative causal genes among our TWAS discoveries. Subsequent enrichment analysis and SMR revealed a significant link between the Golgi apparatus and kidney health, identifying several key GARGs significantly associated with CKD and kidney function. Parallel to transcriptomic analysis, our drug–target MR analysis of the druggable genome identified potential therapeutic targets. This was complemented by PheWAS to explore the potential side effects of drugs targeting these MR-identified genes, ensuring a comprehensive understanding of their safety profile. Molecular docking analysis provided deeper insight, enabling us to identify potentially effective drugs and bridge the gap between genetic findings and therapeutic applications. Moreover, our study benefited from the latest, expansive datasets, analyzing the impacts of 1400 circulating metabolites and 91 circulating inflammatory proteins on CKD and kidney function. This broad, multi-omic approach not only illuminated the complex biology underpinning CKD but also generated valuable insights for future research aimed at understanding and treating this complex disease. Overall, our comprehensive analysis provides valuable insights for researchers seeking to further investigate CKD and kidney function, laying the groundwork for future pharmacological studies.

Our study also presents several limitations that must be highlighted to comprehend the study’s scope and to guide future research directions. Firstly, our study relies predominantly on data from individuals of European ancestry, which may limit the generalizability of our findings to diverse populations. Incorporating a broader range of genetic diversity in future studies could significantly enhance the applicability and relevance of the results. Secondly, while our cross-tissue gene expression approach is innovative, it may not capture the expression weights of all genes relevant to CKD and kidney function. This limitation suggests that further refinement and expansion of gene expression analyses are required. Furthermore, MR analyses, although powerful for inferring causality, have inherent limitations, such as the assumption of no pleiotropy and the accuracy of genetic models used. These limitations necessitate a cautious interpretation of MR findings. In addition, our SMR and drug–target MR analysis primarily focused on cis-eQTLs to understand genetic regulation near gene loci. While this approach provides clear insights into identifying gene targets for CKD, it misses the broader genetic interactions captured by trans-eQTLs. Future research should include both cis- and trans-eQTL data to offer a more comprehensive view of genetic regulation, potentially uncovering novel therapeutic targets for more effective CKD treatments. In our study, we identified genetic associations with the renal function markers eGFRcrea, eGFRcys, and BUN, enriching our understanding of CKD genetics. While these associations provide insights into the physiological processes that may regulate biomarker levels, such as creatinine and cystatin C metabolism, they do not necessarily confirm direct involvement in CKD. Similarly, genetic variations linked to BUN might reflect differences in urea production or clearance mechanisms that are not yet fully understood in the context of CKD. Therefore, these genetic loci associated with renal markers may require further research to determine their roles in the development and progression of CKD. Moreover, all our findings, including high-confidence genes and potential therapeutic targets, should serve as a foundation for generating hypotheses. They require functional validation through experimental studies to confirm their roles in CKD pathophysiology and the efficacy of proposed drug candidates.

## 4. Materials and Methods

A study overview is presented in Figure 1, including data sources, research objectives, methods, and subsequent analyses. All MR analyses in this study are reported following the MR Strengthening the Reporting of Observational Studies in Epidemiology (STROBE) guidelines [31].

### 4.1. Data Sources for CKD and Kidney Functions

Summary-level GWAS data correlating with CKD and kidney functions were sourced from meta-analyses conducted on European ancestry GWASs by the CKDGen Consortium (https://ckdgen.imbi.uni-freiburg.de, accessed on 11 December 2023) [61]. The CKDGen Consortium is an international collaborative effort dedicated to investigating the genetic underpinnings of kidney function and CKD. It involves population-based studies with global participation, focusing on CKD-classifying quantitative traits, particularly GFR and albuminuria. The consortium’s publicly available results foster global scientific collaboration and advance research in the field. The GWAS data used for analysis had already been comprehensively adjusted for sex, age, and multiple covariates, as detailed in the original sources of the data. This comprehensive dataset includes:

1. CKD in European ancestry individuals including 41,395 cases and 439,303 controls [4];
2. eGFRcrea in European ancestry individuals including 567,460 participants [4];
3. eGFRcys in European ancestry individuals including 460,826 participants [5];
4. BUN in European ancestry individuals including 416,178 participants [4];
5. UACR in European ancestry individuals including 547,361 participants [6].

### 4.2. Gene Expression Weights for TWAS

In our TWAS, we aimed to translate SNP associations with CKD and kidney function into gene transcript associations across various tissues, acknowledging the cross-tissue nature of these traits. For this purpose, we utilized cross-tissue gene expression weights generated with sCCA, derived from the GTEx v8 eQTL atlas [13,14].

### 4.3. Cross-Tissue TWAS Analyses

We employed the TWAS/Fusion pipeline to investigate the genetic influences on CKD and kidney-related traits [18]. The GWAS summary statistics were formatted using the munge_sumstats.py script from the LDSC toolkit to ensure compatibility with the FUSION pipeline. Using the FUSION pipeline with default settings, we imputed transcriptomes relevant to our study outcomes, restricting the analysis to autosomal chromosomes. Through FUSION, we accomplished the following:

1. Identified cis-heritable, cross-tissue gene expression features, excluding those from sex chromosomes;
2. Created SNP-based linear predictors of expression levels using our gene expression weights;
3. Computed TWAS test statistics using these predictors and GWAS summary-level Z scores.

The optimal gene expression model was selected by comparing out-of-sample R^2^ values among several models, including penalized linear regressions and Bayesian sparse linear mixed models. To adjust for multiple comparisons and allow for follow-up analysis on a manageable number of findings, we set a Bonferroni-corrected significance threshold at *p* < 1.32 × 10^−6^ (0.05/37,917 cross-tissue sCCA features).

### 4.4. Joint/Conditional Tests, Permutation Tests, and Colocalization Analyses

To assess the robustness of the gene transcript–trait associations identified by TWAS, we initially utilized the FUSION suite’s conditional tests. These tests determined whether multiple TWAS significant sCCA features within a given locus (±500 kb) independently correlated with our kidney-related outcomes, or if they merely reflected artifacts of a single feature–trait association, attributable to correlated expression among features [18]. Joint/conditional analyses utilized GWAS associations remaining after accounting for the predicted expression of other sCCA features within a specific chromosomal locus to estimate the conditionally independent effect of each feature of interest. We set conditional significance at a *p*-value of 0.05. Additionally, to evaluate whether the significance of our gene expression features was driven by strong GWAS signals, we performed permutation tests for each locus, designed to halt after 100,000 permutations, with significance set at a *p*-value of 0.05. Notably, the permutation testing statistic is highly conservative, potentially causing truly causal genes to fail the test [18]. Finally, we conducted colocalization analysis of our TWAS significant genes using the R package coloc (version 5.1.0.1) [20] implemented in FUSION. This analysis aimed to identify evidence of a shared causal variant between the gene expression feature and our trait. We defined strong evidence for colocalization as a PP.H4 > 0.8.

### 4.5. Fine-Mapping of TWAS Associations and High-Confidence Findings

We applied fine-mapping of causal gene sets (FOCUS) to identify genes that likely drove the TWAS gene–trait association signals within specific loci, potentially exerting causal effects on kidney-related traits [19]. FOCUS employs a Bayesian framework that considers LD, TWAS prediction weights, and the effects of pleiotropic SNPs to define gene sets that probabilistically include a causal gene with 90% confidence. Uniquely, this model calculates posterior inclusion probabilities (PIPs) for individual features, with a PIP greater than 0.5 indicating the feature as the most likely causal feature within a risk region [62]. Diverging from conventional colocalization methods, FOCUS excels in scenarios where multiple causal variants or genes are implicated within a locus for a specific trait. In our study, genes with a PIP > 0.5, alongside significant TWAS and conditional analysis *p*-values, were classified as high-confidence genes, suggesting their probable causal role in influencing kidney-related traits.

### 4.6. Cell Type and Tissue Enrichment Analysis

To investigate the expression of high-confidence TWAS genes in various cells and tissues, we utilized the CellMarker database and performed enrichment analysis using the Enrichr tool [63,64]. The CellMarker database is a manually curated resource containing a comprehensive list of cell markers for different cell types in human and mouse tissues. The database includes data from over 100,000 published papers, recording 13,605 cell markers of 467 cell types in 158 human tissues/sub-tissues and 9148 markers of 389 cell types in 81 mouse tissues/sub-tissues. Integrating these data with our enrichment analysis, we identified the specific cell types and tissues where our TWAS-identified genes are significantly expressed, providing critical insights into their potential biological functions and relevance in different cellular contexts.

### 4.7. Gene Set Enrichment Analysis

To elucidate the biological functions and pathways associated with our high-confidence genes identified in the TWAS analysis, we executed a gene enrichment analysis. This analysis was performed utilizing the clusterProfiler R package, version 4.6.2, a tool selected for its robust capabilities in analyzing and visualizing statistical enrichment of gene clusters [65]. Our analysis leveraged gene sets across three domains from the Gene Ontology (GO) database: biological processes, molecular functions, and cellular components [66]. Additionally, we included gene sets from the KEGG and Reactome databases to ensure a comprehensive exploration of the associated biological pathways [67,68]. To focus on gene sets of a manageable size and likely relevance, only gene sets containing between 10 and 1000 genes were included in our analysis.

### 4.8. SMR Analysis of GARGs in CKD and Kidney Function

SMR was employed to estimate the association of GARGs with CKD and kidney function [69]. The list of GARGs was identified through the “GOCC_GOLGI_APPARATUS” gene set using Gene Set Enrichment Analysis (GSEA) in the MSigDB database (http://www.gsea-msigdb.org/gsea/msigdb/index.jsp, accessed on 9 March 2024), including 1634 genes [70]. The eQTL data for these genes were obtained from the eQTLGen Consortium, which contains a comprehensive database of eQTLs identified in blood samples from a large cohort, comprising predominantly 31,684 European individuals [71]. To generate eQTL instruments for GARGs, cis-eQTLs located within 1000 kb on either side of the coding sequence and with a significance threshold of *p* < 5 × 10^−8^ were selected.

The SMR and HEIDI tests were conducted using the SMR software tool (version 1.3.1), with the FDR adjusted for multiple testing corrections employing the Benjamini-Hochberg method. Significant associations were identified based on an FDR-adjusted *p*-value < 0.1 and a HEIDI *p*-value > 0.01. After completing the primary SMR analyses, sensitivity analyses were conducted with the TwoSampleMR R package. The Wald ratio method was used to calculate MR estimates for instruments with a single SNP, whereas the IVW method was applied for instruments with two SNPs. The IVW, MR-Egger, and weighted median methods were employed for proposed instruments comprising more than two variants. For significant genes, colocalization analysis was conducted using the coloc R package. A PP.H4 exceeding 0.8 was considered robust evidence of colocalization, indicating a shared genetic foundation for the association between GARG expression and CKD or kidney function.

### 4.9. Drug–Target MR

The eQTL data for this analysis were obtained from the eQTLGen Consortium [71]. The list of druggable genes was informed by a previous study, including 1375 genes currently under investigation as protein therapeutic targets, 646 genes associated with established drug targets and compounds, and an additional 2281 genes linked to key drug target families [24]. Considering that cis-eQTLs are typically in close proximity to the gene of interest in drug development studies, we selected cis-eQTLs located within a ±100 kb range of each gene’s genomic locus. Through this process, we obtained eQTLs for 2664 druggable genes, subsequently used as exposure instruments in our drug–target MR analyses.

We employed the TwoSampleMR R package (version 0.5.6) for drug–target MR analyses [72]. The initial step involved rigorous filtration of genetic instruments to ensure their high quality. Specifically, SNPs demonstrating weak instrumental strength (F-statistic < 10) were excluded, and conditionally independent SNPs without LD (r^2^ < 0.1, based on the 1000 Genomes European reference panel) were selected as instrumental variables [73]. Furthermore, SNPs implying greater variance in the analyzed outcomes than in the exposure using Steiger filtering were discarded [74].

In the main analysis, the Wald ratio method was used to calculate MR estimates for instruments with a single SNP, whereas the IVW method was applied for instruments with two SNPs. The IVW, MR-Egger, and weighted median methods were employed for proposed instruments comprising more than two variants. The MR-Egger intercept test, especially for instruments with more than two variants, was conducted to evaluate potential pleiotropy in the associations between exposure and outcomes [75]. Cochran’s Q test was performed using both the IVW and MR-Egger methods to assess heterogeneity among Wald ratios (Supplementary Table S16). In cases of significant heterogeneity (*p* < 0.05), the random-effects IVW approach was employed [76]. To adjust for multiple testing, we utilized the Bonferroni correction method, considering *p*-values below 1.90 × 10^−5^ (*p* = 0.05/2644) as significant. For significant genes, a colocalization analysis was conducted using the coloc R package. A posterior probability PP.H4 > 0.80 was used to denote significant colocalization, identifying strongly colocalized genes as potential drug targets.

### 4.10. Phenome-Wide Association Study (PheWAS)

To further assess the horizontal pleiotropy and ascertain potential side effects of the potential drug targets, we conducted a PheWAS analysis utilizing the AstraZeneca PheWAS Portal (https://azphewas.com/, accessed on 21 February 2024) [26]. This portal combines exome sequencing data and phenotypic information from the UK Biobank, encompassing approximately 500,000 participants, providing a powerful resource for extensively analyzing gene–phenotype associations. The portal enabled us to examine potential connections between rare protein-coding variants and a wide range of phenotypes, including 17,361 binary phenotypes and 1419 quantitative phenotypes. This unique opportunity allowed us to uncover novel gen–phenotype relationships and delve into gene multifunctionality. The comprehensive methodological approach employed in this study, including data preparation and analysis procedures, is documented in detail in the source article [26]. To mitigate the risk of false positives, we applied multiple correction techniques and adhered to a significance threshold of 1 × 10^−8^, in line with the default setting on the AstraZeneca PheWAS Portal.

### 4.11. Candidate Drug Prediction

To ascertain the potential of target genes as viable drug targets through assessing protein–drug interactions, we utilized the Drug Signatures Database (DSigDB, http://dsigdb.tanlab.org/DSigDBv1.0/, accessed on 28 March 2024) [77]. DSigDB, containing 22,527 gene sets, 17,389 distinct compounds, and covering 19,531 genes, serves as an extensive repository linking medications and chemicals to their target genes. The identified drug target genes were uploaded to DSigDB to predict drug candidates and evaluate the therapeutic potential of these genes.

### 4.12. Molecular Docking

To elucidate the interactions between drug targets and their corresponding genes, molecular docking analysis was conducted using AutoDock Vina (version 1.2.5) [78]. The three-dimensional structures of proteins encoded by the target genes were predominantly sourced from the RCSB Protein Data Bank (PDB, https://www.rcsb.org/, accessed on 25 February 2024), a comprehensive repository of experimentally determined macromolecular structures. However, for NEK4 and KLHL24, the structures predicted by the AlphaFold database (https://alphafold.ebi.ac.uk/, accessed on 26 February 2024) were utilized due to the absence of experimentally determined structures [79,80]. The structures of drug molecules were acquired from the PubChem database (https://pubchem.ncbi.nlm.nih.gov/, accessed on 25 February 2024) or PDB [81]. Following the preprocessing of structural data with AutoDock Vina, the grid box was configured to either encompass the entire protein or specifically the active site, contingent upon whether an explicit active site was identified for the target. Molecular docking was performed to simulate potential binding scenarios, with results demonstrating binding energies below −5.0 kcal/mol visualized using PyMOL (http://www.pymol.org/, accessed on 26 February 2024). This visualization highlighted significant interactions and binding conformations, underscoring the therapeutic relevance of the drug–gene associations examined. This targeted approach allowed for a nuanced exploration of drug–target interactions, providing valuable insights into their potential efficacy and specificity.

### 4.13. PWMR Analysis

To explore the potential causal links between inflammatory proteins and both CKD and kidney function-related traits, we conducted PWMR analysis. Utilizing protein quantitative trait locus (pQTL) data for 91 circulating inflammatory proteins from previous research [16], we accessed comprehensive GWAS summary statistics for each protein from the University of Bristol (https://www.phpc.cam.ac.uk/ceu/proteins, accessed on 3 February 2024) and the EBI GWAS Catalog (accession numbers GCST90274758 to GCST90274848). We defined a ±1000 kb window around the gene encoding each protein to extract relevant pQTL summary statistics. cis-pQTLs data were selected at a conventional threshold of *p* < 5 × 10^−8^, with instrumental variables being conditionally independent SNPs showing no LD (r^2^ < 0.1, according to the 1000 Genomes European reference panel).

The MR analysis was conducted using the TwoSampleMR R package. For single-SNP instruments, the Wald ratio method was applied for MR estimation. For instruments with two SNPs, we employed the IVW method, and for those with more than two variants, we utilized the IVW, MR-Egger, and weighted median methods. The MR-Egger intercept test was specifically performed for instruments with more than two variants to evaluate potential pleiotropy. Heterogeneity among Wald ratios was assessed using Cochran’s Q test via both IVW and MR-Egger methods. Adjustments for multiple testing significance thresholds were made using the Bonferroni correction, setting *p*-values below 8.06 × 10^−4^ (*p* = 0.05/62) as significant for CKD and eGFRcrea and *p*-values below 8.19 × 10^−4^ (*p* = 0.05/61) as significant for eGFRcys, BUN, and UACR. The coloc R package was utilized for colocalization analysis of MR-significant associations, with a posterior probability PP.H4 > 0.80 indicating significant colocalization. This comprehensive approach enabled us to meticulously investigate the causal relationships between inflammatory proteins and kidney health, yielding insights with significant clinical and therapeutic potential.

### 4.14. Metabolome-Wide MR

To investigate the potential causal links of plasma metabolites with CKD and kidney-related traits, we conducted a comprehensive MR analysis. We drew on GWAS summary statistics from prior research on 1400 metabolites and ratios [58], sourced from the GWAS catalog (https://www.ebi.ac.uk/gwas/, accessed on 10 February 2024) with accession numbers spanning from GCST90199621 to GCST90201020. SNPs associated with metabolites at a genome-wide significance level (*p* < 5 × 10^−8^) and with no LD with other SNPs (r^2^ < 0.1) were selected as instrumental variables. For metabolite-associated SNPs absent in the outcome data, we chose proxy SNPs (r^2^ > 0.8) based on the 1000 Genomes European reference panel [73], ensuring a thorough representation of genetic variations affecting metabolite levels. The MR analysis was conducted using the TwoSampleMR R package. We applied the Wald ratio method for single-SNP instruments, and the IVW method for dual-SNP instruments. Moreover, we applied the IVW, MR-Egger, and weighted median methods for instruments with more than two variants. The MR-Egger intercept test assessed potential pleiotropy in multi-variant instruments, while Cochran’s Q test using IVW and MR-Egger methods evaluated heterogeneity among Wald ratios. Genetic variants associated with more than one metabolite were removed to minimize confounding. Metabolites and ratios exceeding Bonferroni-corrected *p*-values of 5.94 × 10^−5^ for CKD, 6.25 × 10^−5^ for eGFRcrea, 6.52 × 10^−5^ for eGFRcys, 6.26 × 10^−5^ for BUN, and 6.20 × 10^−5^ for UACR were deemed significantly causally related to kidney traits. This rigorous MR approach, leveraging extensive GWAS summary statistics and robust statistical methods, facilitated a nuanced analysis of the genetic factors underlying plasma metabolite associations with CKD and kidney function.

## 5. Conclusions

In conclusion, our study advances the understanding of the genetic basis underlying CKD and kidney function through a comprehensive multi-omic analysis, employing advanced methodologies such as cross-tissue TWAS, fine-mapping, SMR, MR, and molecular docking. This multifaceted approach uncovered key genetic factors and potential therapeutic targets closely associated with CKD and kidney function, expanding our understanding of CKD’s genetic underpinnings and revealing new avenues for its prevention, diagnosis, and treatment. Moreover, by integrating metabolome-wide and protein-wide analyses, our study further illuminates the complex interplay among genetic factors, metabolites, and inflammatory proteins, thereby opening novel strategies for CKD diagnosis and therapy. Overall, our study provides valuable contributions to the field of CKD and kidney function genetics, establishing a robust foundation for developing future clinical interventions.

## Figures and Tables

**Table 1:** High-confidence genes associated with CKD and kidney function (TWAS significant, conditionally significant, and PIP > 0.5).

Trait	Gene	TWAS Z-Score	FOCUS PIP	Joint *p* Value (Conditional Analysis)	PP.H4	Permutation Test *p* Value
CKD	ENSG00000279821	−7.03	0.957	2.10 × 10^−12^	0.947	3.41 × 10^−2^
	PABIR1	−6.01	1	1.80 × 10^−9^	0.986	1.85 × 10^−3^
	RICTOR	−5.80	1	6.60 × 10^−9^	0.937	2.53 × 10^−2^
	MAP3K11	−5.76	0.612	3.30 × 10^−5^	0.968	2.78 × 10^−2^
	DIP2C	−5.04	1	4.60 × 10^−7^	0.976	7.52 × 10^−3^
eGFRcrea	RPL19	−15.82	1	6.00 × 10^−9^	0.854	5.64 × 10^−4^
	KDM5A	−10.99	1	1.50 × 10^−6^	0.971	0
	SGSM3	9.92	1	5.90 × 10^−17^	0.96	9.49 × 10^−4^
	DNAJC16	−9.88	0.998	9.10 × 10^−13^	0.996	0
	PHTF2	9.66	1	4.50 × 10^−22^	0.851	2.53 × 10^−2^
eGFRcys	FUBP1	−9.58	1	3.20 × 10^−17^	1	0
	PRMT6	−7.10	1	1.20 × 10^−12^	0.989	9.01 × 10^−3^
	CSF1	8.80	1	1.40 × 10^−18^	1	2.11 × 10^−4^
	DPYD	−5.15	0.998	2.60 × 10^−07^	0.917	4.17 × 10^−2^
	NR1I3	−5.89	0.827	3.90 × 10^−09^	0.952	2.12 × 10^−3^
BUN	THBS3	13.60	1	1.50 × 10^−27^	0.997	0
	MUC1	13.53	1	1.90 × 10^−33^	0.954	0
	NTN5	10.14	0.704	3.80 × 10^−24^	0.984	8.00 × 10^−5^
	MIER1	−8.21	1	2.20 × 10^−16^	0.983	2.10 × 10^−2^
	NCK1-DT	−8.07	0.975	1.50 × 10^−7^	0.976	1.28 × 10^−2^
UACR	MUC1	−6.83	0.976	1.60 × 10^−4^	0.996	1.31 × 10^−2^
	GGCX	−7.58	1	3.50 × 10^−14^	0.894	8.00 × 10^−4^
	RPL12P16	−7.12	0.992	4.10 × 10^−4^	0.913	5.40 × 10^−3^
	PRKCI	−5.08	0.997	3.80 × 10^−7^	0.999	7.26 × 10^−4^
	IRF1	6.47	0.929	9.60 × 10^−11^	0.899	3.23 × 10^−3^

High-confidence results from TWAS analyses. The top 5 most significant associations for each trait are presented here. TWASs utilized cross-tissue expression weights generated from the GTEx V8 release using sCCA. Associations were deemed high-confidence if they met the following criteria: (1) exceeded the Bonferroni threshold of *p* < 1.32 × 10^−6^ (0.05/37,917 cross-tissue sCCA features); (2) showed colocalization (PP.H4 > 0.8); (3) passed a permutation analysis (*p*-value < 0.05); (4) passed conditional testing (joint *p*-value < 0.05); and (5) passed FOCUS fine-mapping (PIP > 0.5). A Permutation test *p*-value of 0 signifies an association significantly stronger than any permutation result, implying a *p*-value < 0.00001.

**Table 2:** SMR identifies GARGs associated with CKD and kidney function.

Trait	Gene	Effect Size (95% CI) ^a^	FDR-Adjusted *P*_SMR_	*P*_HEIDI_	PP.H4
CKD	ATF6B	1.144 (1.067, 1.227)	0.089	0.11	0.85
eGFRcrea	ZDHHC18	0.006 (0.004, 0.008)	1.51 × 10^−5^	0.50	0.93
	WHAMM	−0.007 (−0.010, −0.005)	3.74 × 10^−5^	0.34	0.98
	NFE2L2	0.042 (0.025, 0.059)	1.15 × 10^−4^	0.06	0.97
	ABO	−0.003 (−0.004, −0.002)	1.15 × 10^−4^	0.23	0.88
	PRKCE	−0.015 (−0.021, −0.009)	1.97 × 10^−4^	0.52	1.00
	STK24	−0.009 (−0.014, −0.005)	2.38 × 10^−3^	0.22	0.84
eGFRcys	COG5	0.004 (0.003, 0.005)	5.35 × 10^−12^	0.32	0.87
	PRKCE	−0.025 (−0.033, −0.018)	9.09 × 10^−9^	0.40	1.00
	GLT8D1	−0.021 (−0.030, −0.012)	1.43 × 10^−4^	0.31	0.82
	PJA2	0.005 (0.003, 0.007)	2.21 × 10^−4^	0.73	0.92
	MAPK3	0.004 (0.002, 0.006)	1.11 × 10^−3^	0.84	0.87
	DDHD2	0.010 (0.005, 0.014)	1.39 × 10^−3^	0.90	0.87
BUN	RASIP1	−0.112 (−0.144, −0.079)	1.47 × 10^−8^	0.51	0.99
	MSH6	0.009 (0.005, 0.013)	4.65 × 10^−3^	0.02	0.93
UACR	RASIP1	0.184 (0.123, 0.244)	9.66 × 10^−7^	0.19	0.97

Results from our SMR analysis. Genes showing significant associations with the trait, as determined with FDR-adjusted *P*_SMR_ < 0.1 and a *P*_HEIDI_ > 0.01, and evidence of colocalization with PP.H4 > 0.8 is displayed. ^a^ Effect size represents the odds ratio (OR) for CKD and the beta coefficient (β) for all other traits. 95% CI: 95% confidence intervals.

**Table 3:** MR identifies druggable genes associated with CKD and kidney function.

Trait	Gene	Method	Effect Size (95% CI) ^a^	*p* Value	PP.H4
CKD	MAP3K11	IVW ^b^	1.102 (1.067, 1.139)	6.85 × 10^−9^	0.891
eGFRcrea	STK36	IVW	−0.009 (−0.012, −0.005)	1.21 × 10^−6^	0.963
	IMPDH2	Wald ratio	−0.027 (−0.038, −0.016)	1.03 × 10^−6^	0.973
	UCN	Wald ratio	0.040 (0.022, 0.057)	6.90 × 10^−6^	0.952
	LAMC1	IVW	0.002 (0.001, 0.003)	4.74 × 10^−7^	0.834
	NRG4	Wald ratio	−0.038 (−0.047, −0.028)	1.48 × 10^−14^	0.813
	POR	IVW	0.003 (0.002, 0.005)	1.23 × 10^−5^	0.888
	GPX1	IVW	−0.019 (−0.025, −0.013)	4.57 × 10^−10^	0.963
	S × 10MA4B	IVW	−0.006 (−0.008, −0.003)	1.52 × 10^−5^	0.957
	KCNMA1	IVW	0.004 (0.003, 0.005)	5.21 × 10^−13^	0.917
	KBTBD2	IVW	0.003 (0.002, 0.005)	1.34 × 10^−5^	0.915
	ACVR2A	IVW	−0.009 (−0.013, −0.005)	8.57 × 10^−7^	0.866
	MAST3	IVW	−0.006 (−0.008, −0.003)	9.11 × 10^−6^	0.956
	CASP9	IVW	−0.008 (−0.010, −0.005)	1.99 × 10^−11^	0.927
	BTN3A2	IVW	0.002 (0.001, 0.003)	6.34 × 10^−6^	0.882
	CA3	Wald ratio	0.038 (0.022, 0.053)	2.21 × 10^−6^	0.967
	ITIH4	IVW	0.004 (0.003, 0.005)	1.32 × 10^−18^	0.979
eGFRcys	STK36	IVW	−0.017 (−0.024, −0.010)	1.07 × 10^−6^	0.891
	IMPDH2	Wald ratio	−0.031 (−0.044, −0.017)	9.49 × 10^−6^	0.963
	CHRNB1	IVW	−0.006 (−0.009, −0.003)	1.59 × 10^−5^	0.916
	MANBA	IVW	0.017 (0.014, 0.020)	2.15 × 10^−29^	0.993
	KLHL24	IVW	−0.006 (−0.007, −0.004)	5.67 × 10^−13^	0.851
	GPX1	IVW	−0.027 (−0.035, −0.019)	1.07 × 10^−10^	0.924
	MGMT	IVW	−0.003 (−0.004, −0.002)	5.81 × 10^−9^	0.875
	PSMB10	IVW	−0.023 (−0.031, −0.015)	1.63 × 10^−8^	0.972
	FKBP5	Wald ratio	−0.049 (−0.071, −0.028)	9.49 × 10^−6^	0.894
	LNPEP	IVW	0.009 (0.006, 0.011)	1.01 × 10^−9^	0.906
	PPIA	IVW	0.011 (0.008, 0.014)	3.73 × 10^−13^	0.970
	NEU1	IVW	0.039 (0.031, 0.047)	4.90 × 10^−20^	0.981
	TOP2A	Wald ratio	−0.053 (−0.077, −0.030)	1.08 × 10^−5^	0.887
	CASP9	IVW	−0.007 (−0.009, −0.005)	3.48 × 10^−11^	0.943
BUN	WFIKKN1	IVW	0.012 (0.007, 0.017)	1.18 × 10^−5^	0.946
	NRG4	Wald ratio	0.074 (0.050, 0.097)	6.20 × 10^−10^	0.814
	WNT6	Wald ratio	−0.130 (−0.175, −0.084)	2.77 × 10^−8^	0.972
	LDHA	IVW	−0.017 (−0.024, −0.009)	1.16 × 10^−5^	0.850
	THBS3	IVW	−0.019 (−0.023, −0.014)	6.00 × 10^−16^	1.000
	CASP9	IVW	0.012 (0.008, 0.016)	5.89 × 10^−8^	0.899
UACR	UCN	Wald ratio	0.269 (0.170, 0.368)	9.88 × 10^−8^	0.974
	SLC22A4	IVW	0.028 (0.019, 0.038)	5.86 × 10^−9^	0.945
	NEK4	Wald ratio	−0.167 (−0.230, −0.105)	1.72 × 10^−7^	0.922
	PNMT	Wald ratio	0.180 (0.117, 0.242)	1.50 × 10^−8^	0.990

Genes exhibiting significant association with the trait, passing a Bonferroni threshold of 1.90 × 10^−5^ (*p* = 0.05/2644), and exhibiting colocalization with PP.H4 > 0.8 are displayed. ^a^ Effect size represents the OR for CKD and the beta coefficient for all other traits. ^b^ Inverse variance weighted (IVW).

**Table 4:** Molecular docking results of potential drug candidates with their target proteins.

Target	PDB ID	Drug	PubChem ID	Binding Energy ^b^
CASP9	1JXQ	Capsaicin	1548943	−7.483
LNPEP	4PJ6	Capsaicin	1548943	−6.626
ITIH4	AF-Q14624-F1 ^a^	Capsaicin	1548943	−6.411
LDHA	4ZVV	Capsaicin	1548943	−6.298
GPX1	2F8A	Capsaicin	1548943	−6.245
CA3	3UYQ	Capsaicin	1548943	−5.875
TOP2A	6ZY5	5-Fluorouracil	3385	−5.827
NEK4	AF-P51957-F1 ^a^	5-Fluorouracil	3385	−5.654
KLHL24	AF-Q6TFL4-F1 ^a^	5-Fluorouracil	3385	−5.545
IMDH2	1B3O	5-Fluorouracil	3385	−5.502
LAMC1	AF-P11047-F1 ^a^	5-Fluorouracil	3385	−5.391
MGMT	1QNT	5-Fluorouracil	3385	−5.312
CASP9	1JXQ	5-Fluorouracil	3385	−5.296
CASP9	1JXQ	L-aspartic acid	5960	−5.066
LDHA	4ZVV	5-Fluorouracil	3385	−5.051
LNPEP	4PJ6	L-aspartic acid	5960	−5.018
CA3	3UYQ	L-aspartic acid	5960	−5.009
LDHA	4ZVV	L-aspartic acid	5960	−4.679
FKBP5	5OMP	5-Fluorouracil	3385	−4.66
GPX1	2F8A	5-Fluorouracil	3385	−4.622
PSMB10	6E5B	5-Fluorouracil	3385	−4.579
ITIH4	AF-Q14624-F1 ^a^	L-aspartic acid	5960	−4.511
GPX1	2F8A	L-aspartic acid	5960	−4.189

^a^ The structures predicted by the AlphaFold database (https://alphafold.ebi.ac.uk/, accessed on 26 February 2024) were utilized due to the absence of experimentally determined structures. ^b^ Binding energy, measured in kcal/mol, quantifies the affinity between each drug and its target, with more negative values indicating stronger binding interactions.

**Table 5:** PWMR identifies inflammatory proteins significantly associated with CKD and kidney function.

Trait	Protein	Method	Effect Size (95% CI) ^a^	*p* Value	PP.H4
CKD	FGF5	IVW	0.908 (0.876, 0.942)	2.25 × 10^−7^	0.976
eGFRcrea	FGF5	IVW	0.005 (0.004, 0.006)	3.54 × 10^−13^	0.958
eGFRcrea	DNER	IVW	−0.005 (−0.008, −0.003)	1.55 × 10^−4^	0.960
eGFRcys	FGF5	IVW	0.004 (0.002, 0.006)	3.05 × 10^−5^	0.995
BUN	FGF5	IVW	−0.007 (−0.009, −0.004)	2.60 × 10^−6^	0.975

Results from our PWMR analysis. Proteins exhibiting a significant association with the trait after Bonferroni adjustment and exhibiting colocalization with PP.H4 > 0.8 are displayed. ^a^ Effect size represents the odds ratio (OR) for CKD and the beta coefficient (β) for all other traits.

## Data Availability

All analyses in this study were conducted using publicly available data. The details of the source datasets are mentioned in the Methods Section.

## Supplementary Materials

The following supporting information can be downloaded at: https://www.mdpi.com/article/10.3390/ijms25116033/s1.
