# Supplementary material for: Multi-Omic Analysis Reveals Genetic Determinants and Therapeutic Targets of Chronic Kidney Disease and Kidney Function
Source: Int J Mol Sci. 2024 May 30;25(11):6033. doi: 10.3390/ijms25116033 (PMC11172763; doi:10.3390/ijms25116033)

Appendix B list

Supplementary Figure S1. Cell type and tissue enrichment analysis for high-confidence TWAS genes.

Supplementary Figure S2: Molecular Docking Analysis of capsaicin with ITH4

Supplementary Figure S3: Molecular Docking Analysis of capsaicin with LDHA

Supplementary Figure S4: Molecular Docking Analysis of capsaicin with GPX1

Supplementary Figure S5: Molecular Docking Analysis of capsaicin with CA3

Supplementary Figure S6: Molecular Docking Analysis of 5-fluorouracil with TOP2A

Supplementary Figure S7: Molecular Docking Analysis of 5-fluorouracil with LAMC1

Supplementary Figure S8: Molecular Docking Analysis of 5-fluorouracil with NEK4

Supplementary Figure S9: Molecular Docking Analysis of 5-fluorouracil with KLHL24

Supplementary Figure S10: Molecular Docking Analysis of 5-fluorouracil with IMDH2

Supplementary Figure S11: Molecular Docking Analysis of 5-fluorouracil with MGMT

Supplementary Figure S12: Molecular Docking Analysis of 5-fluorouracil with CASP9

Supplementary Figure S13: Molecular Docking Analysis of L-aspartic acid with CASP9

Supplementary Figure S14: Molecular Docking Analysis of 5-fluorouracil with LDHA

Supplementary Figure S15: Molecular Docking Analysis of L-aspartic acid with LNPEP

Supplementary Figure S16: Molecular Docking Analysis of L-aspartic acid with CA3

Supplementary Figure S1. Cell type and tissue enrichment analysis for high-confidence TWAS genes. This bar plot showcases the top 10 cell types and tissues with significant gene enrichment. The genes associated with each cell type and tissue are listed alongside their respective bars.

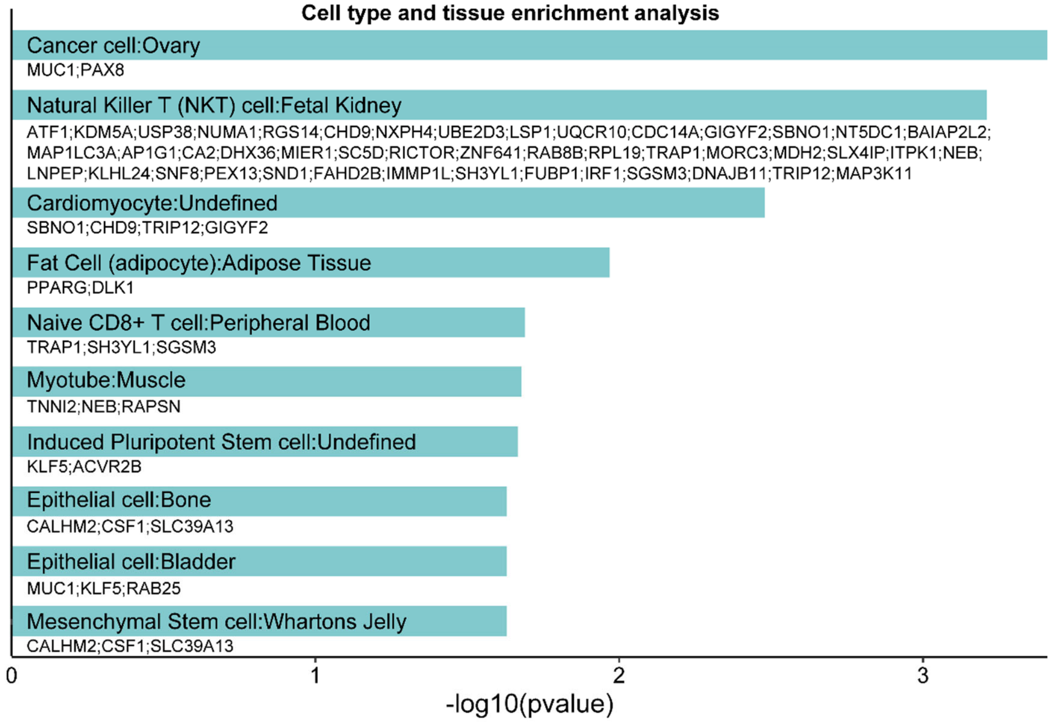

Supplementary Figure S2. Molecular Docking Analysis of capsaicin with ITH4. (Left) The overall structure of ITH4 in complex with capsaicin. (Right) A close-up view of the binding pocket, with the color scheme and symbols corresponding to those in Fig. 5.

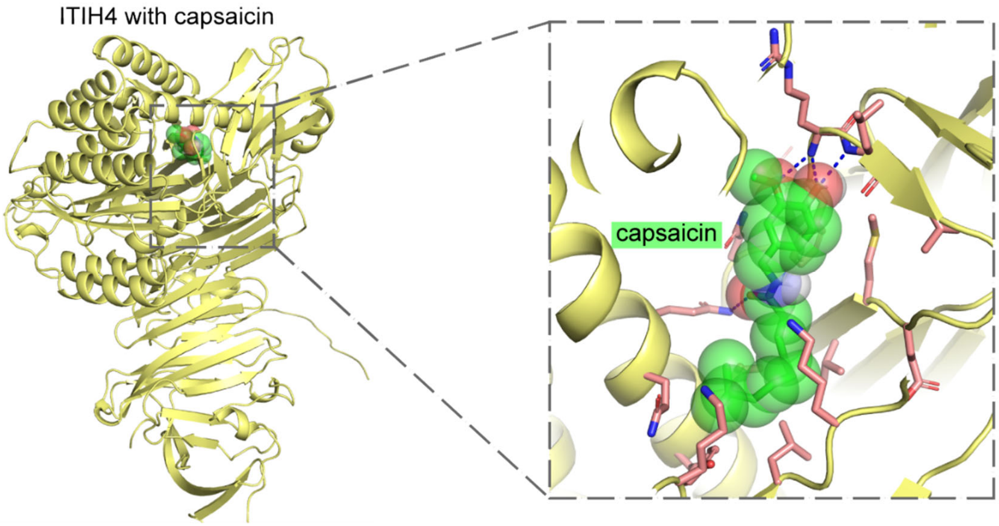

Supplementary Figure S3. Molecular Docking Analysis of capsaicin with LDHA. (Left) The overall structure of LDHA in complex with capsaicin. (Right) A close-up view of the binding pocket, with the color scheme and symbols corresponding to those in Fig. 5.

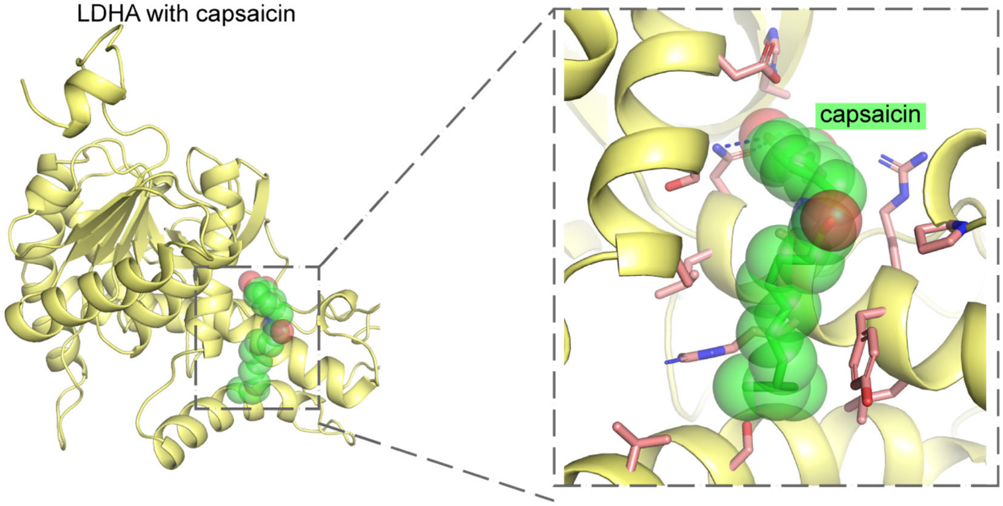

Supplementary Figure S4. Molecular Docking Analysis of capsaicin with GPX1. (Left) The overall structure of GPX1 in complex with capsaicin. (Right) A close-up view of the binding pocket, with the color scheme and symbols corresponding to those in Fig. 5.

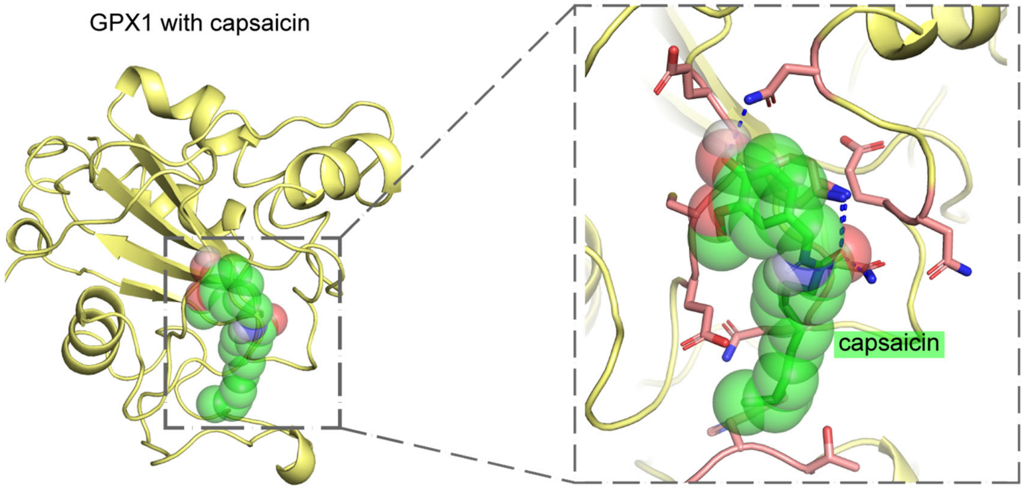

Supplementary Figure S5. Molecular Docking Analysis of capsaicin with CA3. (Left) The overall structure of CA3 in complex with capsaicin. (Right) A close-up view of the binding pocket, with the color scheme and symbols corresponding to those in Fig. 5.

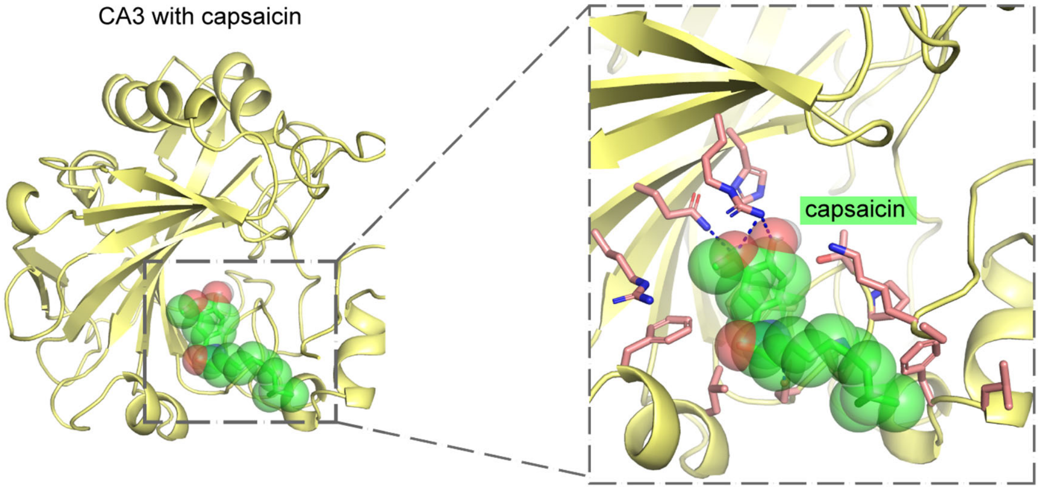

Supplementary Figure S6. Molecular Docking Analysis of 5-fluorouracil with TOP2A. (Left) The overall structure of TOP2A in complex with 5-fluorouracil. (Right) A close-up view of the binding pocket, with the color scheme and symbols corresponding to those in Fig. 5.

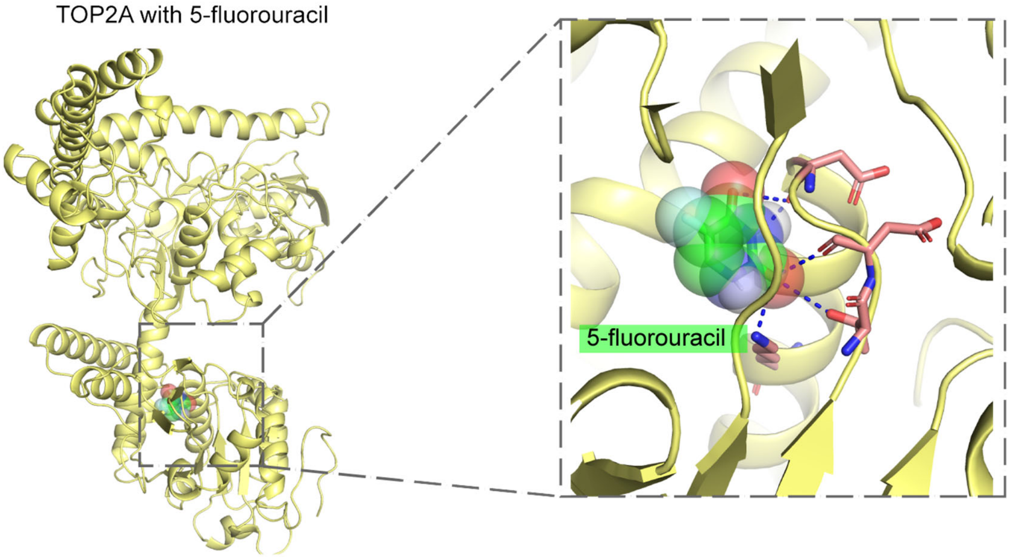

Supplementary Figure S7. Molecular Docking Analysis of 5-fluorouracil with LAMC1. (Left) The overall structure of LAMC1 in complex with 5-fluorouracil. (Right) A close-up view of the binding pocket, with the color scheme and symbols corresponding to those in Fig. 5.

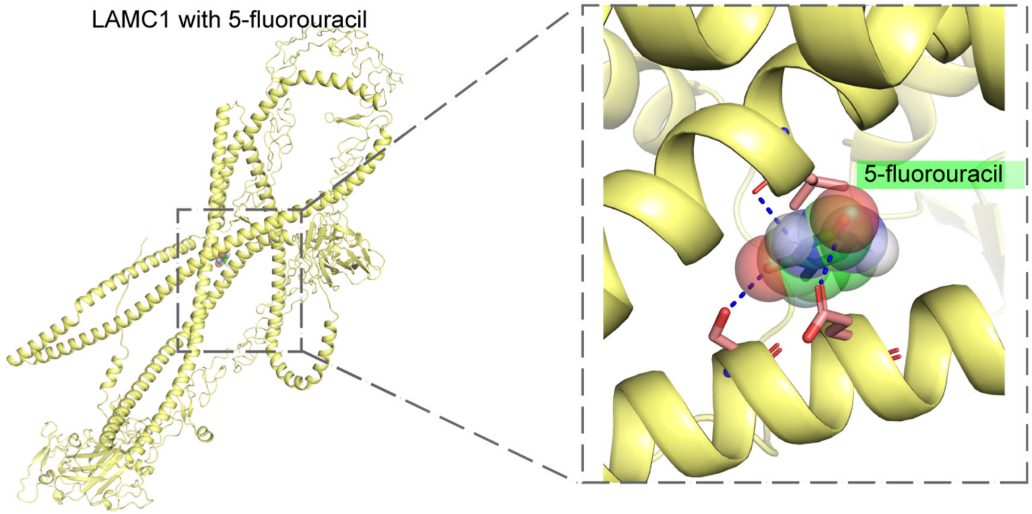

Supplementary Figure S8. Molecular Docking Analysis of 5-fluorouracil with NEK4. (Left) The overall structure of NEK4 in complex with 5-fluorouracil. (Right) A close-up view of the binding pocket, with the color scheme and symbols corresponding to those in Fig. 5.

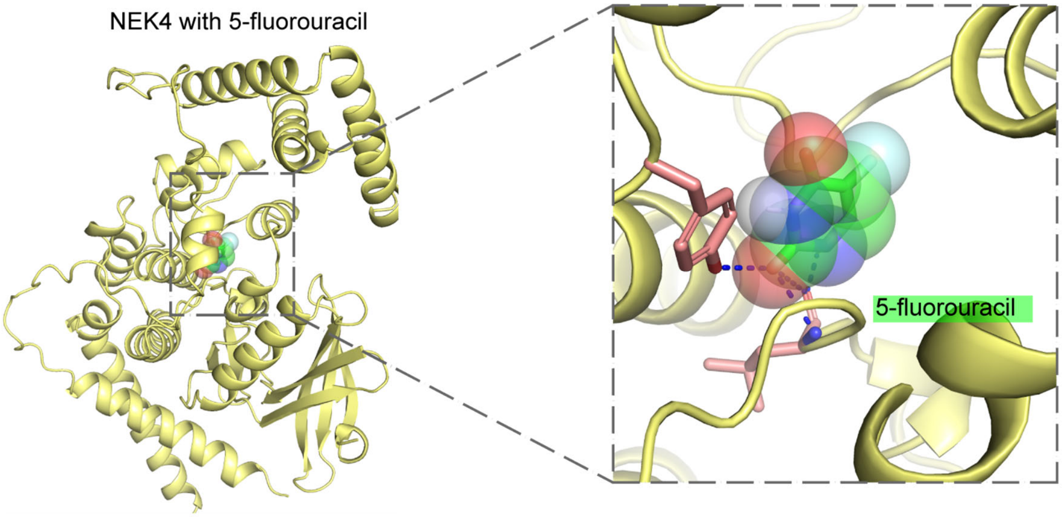

Supplementary Figure S9. Molecular Docking Analysis of 5-fluorouracil with KLHL24. (Left) The overall structure of KLHL24 in complex with 5-fluorouracil. (Right) A close-up view of the binding pocket, with the color scheme and symbols corresponding to those in Fig. 5.

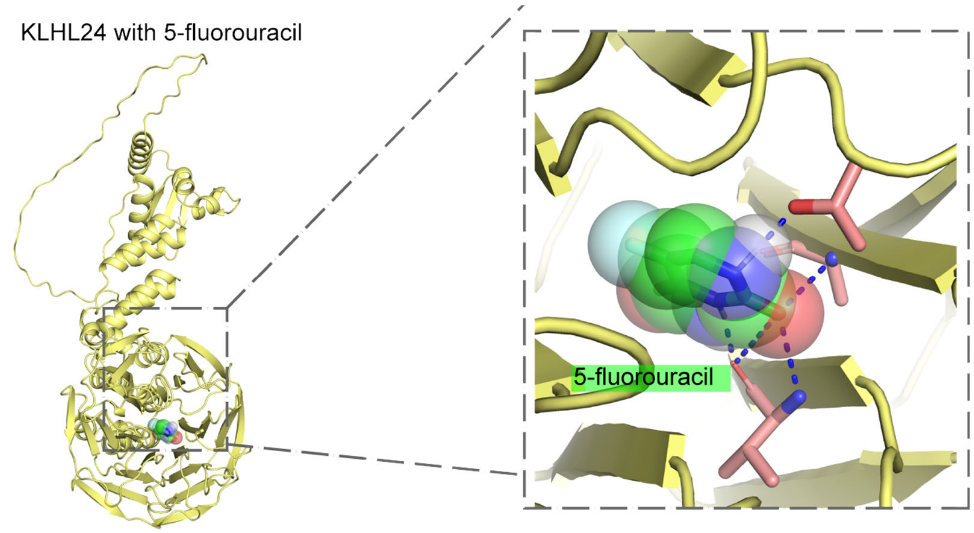

Supplementary Figure S10. Molecular Docking Analysis of 5-fluorouracil with IMDH2. (Left) The overall structure of IMDH2 in complex with 5-fluorouracil. (Right) A close-up view of the binding pocket, with the color scheme and symbols corresponding to those in Fig. 5.

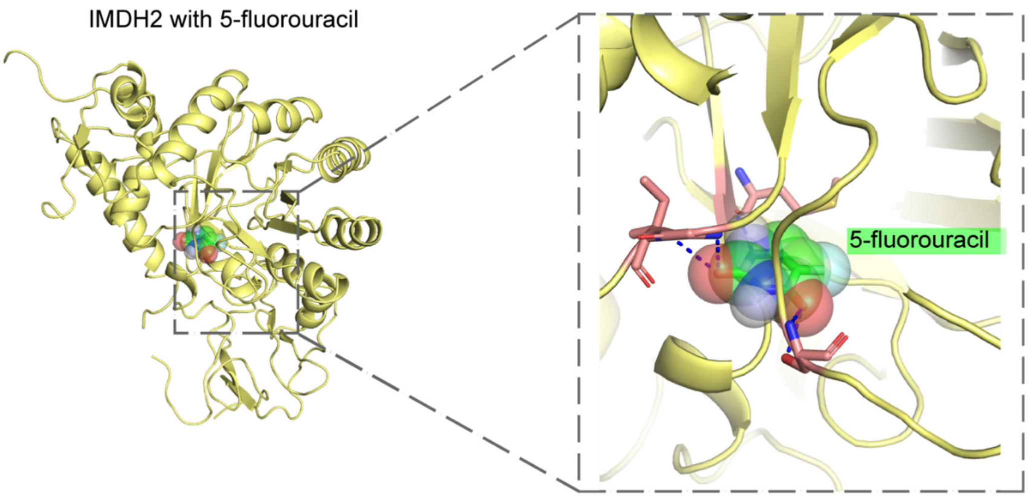

Supplementary Figure S11. Molecular Docking Analysis of 5-fluorouracil with MGMT. (Left) The overall structure of MGMT in complex with 5-fluorouracil. (Right) A close-up view of the binding pocket, with the color scheme and symbols corresponding to those in Fig. 5.

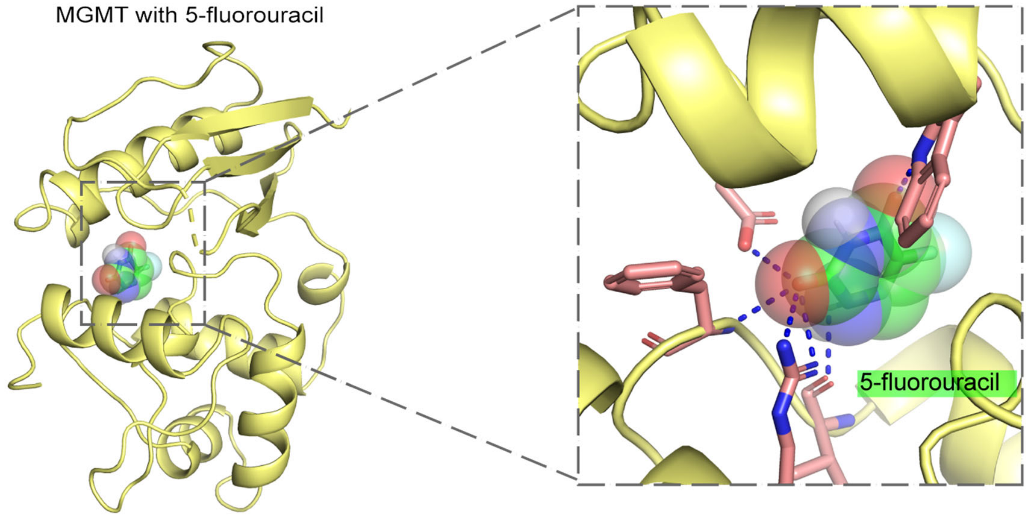

Supplementary Figure S12. Molecular Docking Analysis of 5-fluorouracil with CASP9. (Left) The overall structure of the CASP9 dimer complexed with 5-fluorouracil, with monomer A depicted in yellow and monomer B in cyan. (Right) A close-up view of the binding pocket, with the color scheme and symbols corresponding to those in Fig. 5.

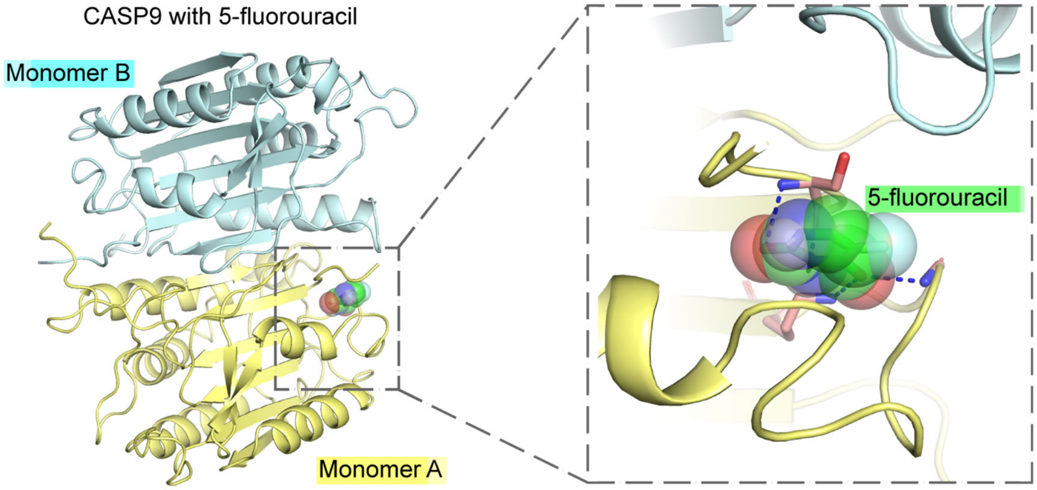

Supplementary Figure S13. Molecular Docking Analysis of L-aspartic acid with CASP9. (left) The overall structure of the CASP9 dimer complexed with L-aspartic, with monomer A depicted in yellow and monomer B in cyan. (Right) A close-up view of the binding pocket, with the color scheme and symbols corresponding to those in Fig. 5.

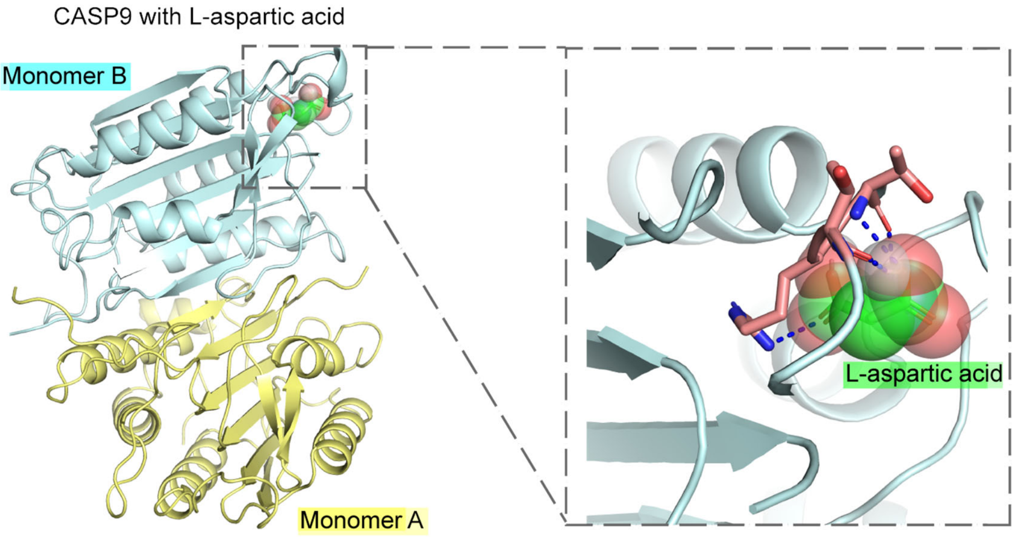

Supplementary Figure S14. Molecular Docking Analysis of 5-fluorouracil with LDHA. (Left) The overall structure of LDHA in complex with 5-fluorouracil. (Right) A close-up view of the binding pocket, with the color scheme and symbols corresponding to those in Fig. 5.

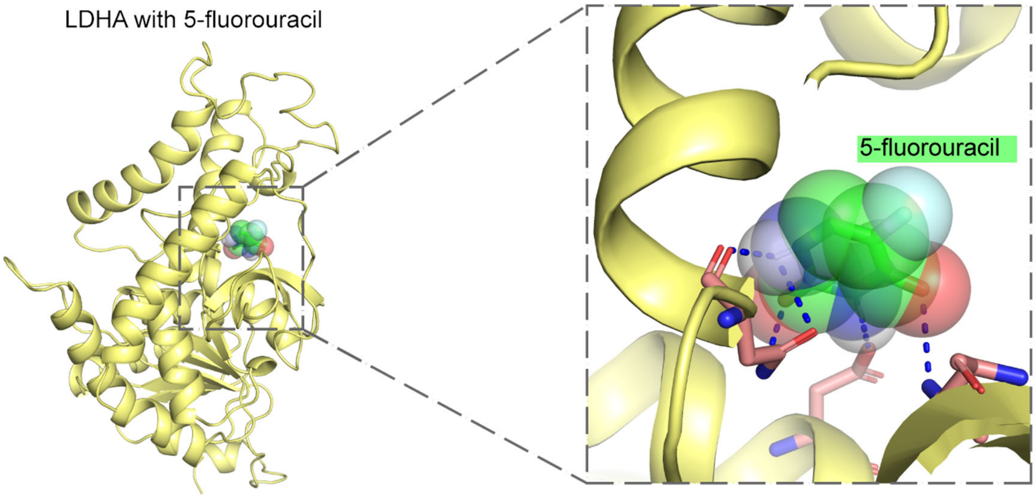

Supplementary Figure S15. Molecular Docking Analysis of L-aspartic acid with LNPEP. (Left) The overall structure of LNPEP in complex with L-aspartic acid. (Right) A close-up view of the binding pocket, with the color scheme and symbols corresponding to those in Fig. 5.

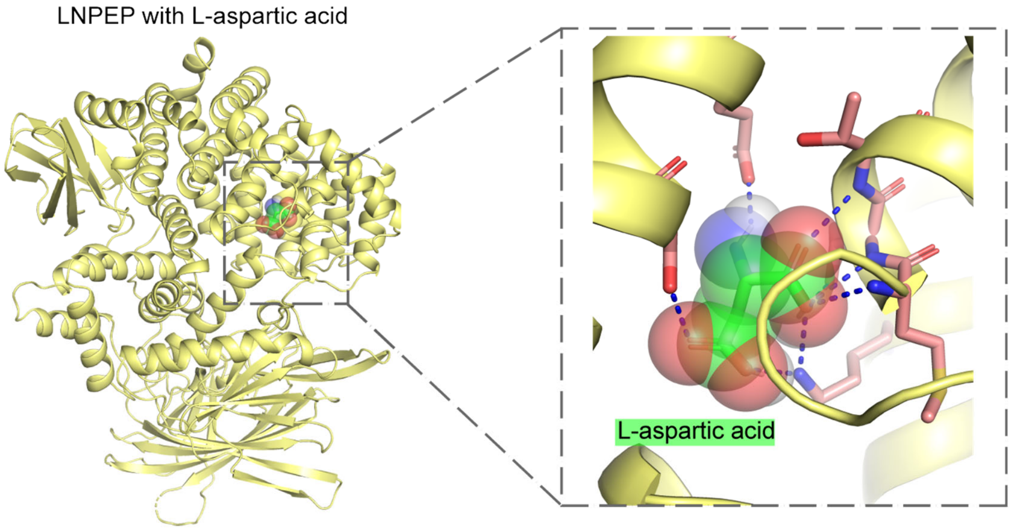

Supplementary Figure S16. Molecular Docking Analysis of L-aspartic acid with CA3. (Left) The overall structure of LNPEP in complex with CA3. (Right) A close-up view of the binding pocket, with the color scheme and symbols corresponding to those in Fig. 5

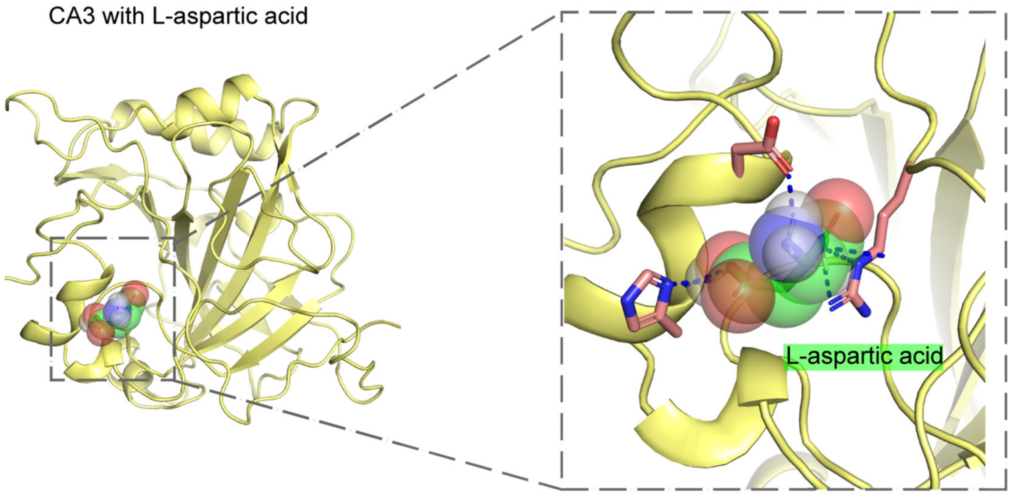

Supplement: Supplementary file 1 [file ijms-25-06033-s001.zip › ijms-2993601-supplementary.pdf]
